# Elucidating the transcriptional program of feline injection-site sarcoma using a cross-species mRNA-sequencing approach

**DOI:** 10.1186/s12885-019-5501-z

**Published:** 2019-04-04

**Authors:** Qi Wei, Stephen A. Ramsey, Maureen K. Larson, Noah E. Berlow, Donasian Ochola, Christopher Shiprack, Amita Kashyap, Bernard Séguin, Charles Keller, Christiane V. Löhr

**Affiliations:** 1 Department of Biomedical Sciences, Oregon State University, Corvallis, OR USA; 2 Department of Clinical Sciences, Oregon State University, Corvallis, OR USA; 3 Children’s Cancer Therapy Development Institute, Beaverton, OR USA; 4 Flint Animal Cancer Center, Colorado State University, Fort Collins, CO USA

**Keywords:** Sarcoma, Comparative oncology, mRNA-seq, Feline, Transcriptome

## Abstract

**Background:**

Feline injection-site sarcoma (FISS), an aggressive iatrogenic subcutaneous malignancy, is challenging to manage clinically and little is known about the molecular basis of its pathogenesis. Tumor transcriptome profiling has proved valuable for gaining insights into the molecular basis of cancers and for identifying new therapeutic targets. Here, we report the first study of the FISS transcriptome and the first cross-species comparison of the FISS transcriptome with those of anatomically similar soft-tissue sarcomas in dogs and humans.

**Methods:**

Using high-throughput short-read paired-end sequencing, we comparatively profiled FISS tumors vs. normal tissue samples as well as cultured FISS-derived cell lines vs. skin-derived fibroblasts. We analyzed the mRNA-seq data to compare cancer/normal gene expression level, identify biological processes and molecular pathways that are associated with the pathogenesis of FISS, and identify multimegabase genomic regions of potential somatic copy number alteration (SCNA) in FISS. We additionally conducted cross-species analyses to compare the transcriptome of FISS to those of soft-tissue sarcomas in dogs and humans, at the level of cancer/normal gene expression ratios.

**Results:**

We found: (1) substantial differential expression biases in feline orthologs of human oncogenes and tumor suppressor genes suggesting conserved functions in FISS; (2) a genomic region with recurrent SCNA in human sarcomas that is syntenic to a feline genomic region of probable SCNA in FISS; and (3) significant overlap of the pattern of transcriptional alterations in FISS with the patterns of transcriptional alterations in soft-tissue sarcomas in humans and in dogs. We demonstrated that a protein, BarH-like homeobox 1 (BARX1), has increased expression in FISS cells at the protein level. We identified 11 drugs and four target proteins as potential new therapies for FISS, and validated that one of them (GSK-1059615) inhibits growth of FISS-derived cells in vitro*.*

**Conclusions:**

(1) Window-based analysis of mRNA-seq data can uncover SCNAs. (2) The transcriptome of FISS-derived cells is highly consistent with that of FISS tumors. (3) FISS is highly similar to soft-tissue sarcomas in dogs and humans, at the level of gene expression. This work underscores the potential utility of comparative oncology in improving understanding and treatment of FISS.

**Electronic supplementary material:**

The online version of this article (10.1186/s12885-019-5501-z) contains supplementary material, which is available to authorized users.

## Background

Feline injection-site sarcoma (FISS) is an aggressive subcutaneous soft-tissue cancer that occurs in approximately 1 in 10,000 domestic cats, frequently at vaccination sites [1]. The etiology of FISS appears to involve non-resolving local inflammation leading to neoplastic transformation of fibroblasts or myofibroblasts and the development of a tumor mass [2]. The triggers of this inflammation probably include vaccine adjuvants and mechanical insult to the subcutis (with latency estimates ranging from months to years [3]), although predisposing genetic factors for FISS have also been noted [2, 4, 5]. Due to its invasiveness, FISS is difficult to treat; with the current standard of care, radical excision with pre- or post-surgical radiation, recurrence occurs in 15–70% of cases and metastasis in 20% of cats [1, 6, 7]. Average survival may be as short as 6 months [6] but can be as long as 5 years with surgery and radiotherapy [8]. Results from clinical trials of adjuvant doxorubicin suggest some benefit but are neither curative nor tailored to the patient-specific molecular signature of this histologically diverse neoplasm [1], and thus, new therapeutic approaches are needed. In the context of human soft tissue sarcoma, new molecular mechanisms and new therapeutic targets have been discovered through the use of comprehensive gene expression profiling of tumors and normal tissues in case-control studies [9–11]. Additionally, new insights into pathogenic mechanisms and driver mutations have been uncovered through comparative oncology approaches with transcriptome or exome profiling [12–14]. In the context of feline sarcoma, somatic copy number alterations (SCNAs) have been studied genome-wide, revealing recurrent copy number gains and losses [4], but it is unknown to what extent these alterations correlate with elevated or reduced tumor transcript abundances, though such changes would be expected based on current understanding of cancer biology [15]. However, despite the promise of such systems-biology approaches, the tumor transcriptome of FISS has been neither systematically studied nor compared to the transcriptomes of soft tissue sarcomas in other species such as domestic dogs or humans.

In this study, we used high-throughput short-read mRNA sequencing (mRNA-seq) [15–17] to profile the transcriptomes of three FISS tumors and three normal patient-matched tissue samples as well as cultured FISS-derived cell lines and skin fibroblasts. With this approach we identified 3049 transcripts with altered abundance between FISS tumors and normal skin (of which 335 were also differentially expressed in the FISS cell lines vs. cultured fibroblasts), 17 biological processes or molecular pathways whose associated genes are enriched in the set of FISS differentially expressed genes, and nine genomic regions with coherent dysregulation that are probable FISS SCNAs (of which eight are novel per the current state of knowledge [4]). We then analyzed the degree of consistency between the FISS transcriptome and human cancer gene lists and patterns of differential expression in human and dog sarcomas. Among human orthologs of cat genes with altered expression in FISS vs. normal skin, we found significant enrichment of tumor suppressor genes (TSGs) [18] and oncogenes [19]. Additionally, we found a 10 Mbp region of potential FISS SCNA whose human syntenic region has a recurrent SCNA in sarcoma. Using ortholog mapping, we compared the pattern of transcriptional dysregulation in FISS with the transcriptional patterns in soft tissue sarcomas in humans and in dogs. We found significant three-way overlap: 53 genes that are upregulated in sarcoma vs. normal tissue in all three species and 38 genes that are downregulated in sarcoma in all three species. Finally, we analyzed the sets of 53 and 38 genes that are consistently upregulated or downregulated using data from a cell line-based drug-to-transcriptome screen, identifying 11 drugs and four drug targets whose reported effects on cell lines suggest possible therapeutic benefit in treating FISS. This work represents the first mRNA-seq study of a feline neoplasm of which we are aware, and represents a template for how cross-species approaches could be used to study other types of feline cancers. The feline mRNA-seq data from this study are available in a public repository as a resource for the scientific community (see Methods).

## Methods

### Animals

Through the biobank at the Carlson College of Veterinary Medicine at Oregon State University, we obtained samples from three histologically confirmed FISS tissues and patient-matched normal tissues (two skin samples and one skeletal muscle sample) from the three cats aged 10–13 years. The FISS samples were high grade soft tissue sarcomas from neutered male domestic shorthair cats (thigh or between shoulder blades); none of the cats had metastasis at time of surgery. Tumor volumes ranged up to 500 cm^3^. The surgical treatment of FISS patients and the diagnostic work-up of tumor samples followed best clinical practices. Two cats were treated by amputation of the affected leg. In the third cat surgical excision included the dorsal processes of thoracic vertebrae. Clinical follow-up data were not available. Through the biobank at the Flint Animal Cancer Center at Colorado State University, we obtained samples from nine histologically confirmed canine soft tissue sarcomas (four grade 3 soft tissue sarcomas, two grade 3 peripheral nerve sheath tumors, two grade 1 myxomatous soft tissue sarcomas, and one grade 2 soft tissue sarcoma) and matched normal skeletal muscle samples. One of the grade 3 soft tissue sarcomas was annotated in the clinical record as possibly being histiocytic sarcoma given its anatomic location (joint capsule) and lymph node metastasis.

### Cell culture

Feline FISS cell lines (derived from tumors from cat04, cat05, and cat07) were obtained from the College of Veterinary Medicine at the University of Wisconsin (courtesy of David Vail, Michael Huelsmeyer, and Ilene Kurzman). Primary cultures of feline skin fibroblasts were obtained from the biobank at the Carlson College of Veterinary Medicine at Oregon State University. Cells were grown in high glucose Dulbecco’s Modified Eagle’s Medium (DMEM) containing L-glutamine and sodium pyruvate (Corning #10-013CV, Axygen Incorporated, Union City, CA) with 10% (*v*/v) fetal bovine serum (FBS) (HyClone #SH3039603, HyClone Laboratories Inc., Logan, UT), 100 U/mL penicillin, and 100 μg/mL streptomycin (“pen/strep”) (Gibco #15140122, Invitrogen, Carlsbad, CA) at 37 °C and 5% CO_2_. Primary cell cultures were harvested for RNA at third passage and cells were harvested at 70% confluence.

### Annotated genome information

For all computational analyses that used the complete sequence of the cat genome, we used the sequence for genome assembly *Felis catus* 6.2 (Broad Institute, Cambridge, MA, USA; released Sep. 2011) [20], which we downloaded from the Ensembl database (release 87, Dec. 2016). We obtained information about cat gene locations and exon structures in a Gene Transfer Format file from Ensembl (release 87). We obtained gene annotation information via the BioMart tool from Ensembl (release 87). For genome visualization we used the Integrated Genomics Viewer version 2.3.90 [21].

### RNA isolation

We isolated RNA from tissue samples and cell cultures using the Norgen Total RNA Purification Kit #17200 (Norgen Biotek, Thorold, ON), with elution using nuclease-free water.

### FISS mRNA-seq profiling

RNA sample library preparation and high-throughput sequencing were performed by the Genomics Core at the Center for Genome Research and Biocomputing at Oregon State University. RNA samples were rRNA-depleted using Ribo-Zero Gold (Illumina, San Diego, CA, USA); strand-specific mRNA-seq libraries were prepared using the PrepX RNA-seq for Illumina Library kit on the Apollo 324 (Wafergen, Fremont, CA, USA); and barcoded libraries were sequenced on a HiSeq 3000 (Illumina) at 2 × 100 bp (paired-end sequencing) on one lane for the first batch of samples (see Additional file 1: Table S1). We generated sequence quality reports using FASTQC [22] and then aligned the reads to the annotated cat genome using the software tool STAR [23] (in the alignment, only uniquely aligned reads were retained, and we used basic two-pass mapping, with all first-pass junctions inserted into the genome indices). The alignment yielded an average of 1.0 × 10^8^ mapped reads per sample. Next, we obtained counts of aligned reads per gene with featureCounts (version 1.5.1) using the Subread software program [24] with the minimum mapping quality score parameter set to the value 3.0 and genome-wide cat gene and exon annotations from Ensembl Release 87 [25]. Given the fibrosarcoma histotypes of the FISS tumors in this study, for the supervised analysis of differential expression in primary tissue, we compared FISS to normal skin tissue only (not muscle). For testing individual genes for differential expression between the sample groups, we used DESeq2 [26] with the Wald test and with *p-*value adjustment using the Benjamini-Hochberg method [27]. For each gene, we also computed log-scale normalized counts as log_2_(1 + *C*), where *C* is the normalized expression level from DESeq2. We also re-analyzed the mRNA-seq data using the *Felis catus* 9.0 genome assembly and the Ensembl 95 gene annotations; we compared the gene-level FISS/skin log_2_ ratios that we obtained using FelCat9 with the gene-level ratios that we obtained using FelCat6.2; they were correlated at *R* = 0.995.

### Principal component analysis (PCA)

For PCA, we used the function prcomp from the R software package stats ver. 3.4.2. For plotting loading scores we used the R package ggbiplot [28]. We selected high-variance genes with minimum threshold of 2.0 for the variance of the normalized log_2_ expression levels across the samples for the PCA analysis.

### Gene set enrichment analysis

For Gene Set Enrichment Analysis (GSEA) [29], we used the Java JAR file-deployed GSEA software (ver. 2.1.0) in GseaPreranked mode, with genes labeled by official gene symbol and ranked by log_2_(sarcoma/skin) or log_2_(sarcoma cells / fibroblast) as appropriate for the sample type (tissue or cultured cells). For the gene sets, we used the C5 gene sets from the MSigDB database (release 5.2) [29]. For the in silico compound screen, we screened gene sets from our study against ten drug-to-cell-line datasets using the Enrichr web tool (ver. 06/28/2017) [30] with a cutoff of FDR < 0.05. Redundant functional annotations were reduced to representative annotations by manual curation.

### Oncogenes and tumor suppressor genes (TSGs) datasets

We obtained lists of human oncogenes from the ONGene database [19]. Additionally, we obtained lists of tumor suppressor genes from the TSGene database [18]. For the oncogene/TSG analysis, we separated all identified feline genes differentially expressed in tissues into orthologs of human TSGs, oncogenes, or genes that are neither TSGs nor oncogenes. For obtaining a “background” gene list, filtered for all genes whose normalized log_2_ expression count exceeded 2.0 in either the sarcoma or skin sample for cat01.

### qPCR

Reverse Transcription was performed in a 7.5 ng RNA/μL reaction volume using the High Capacity cDNA Reverse Transcription Kit (Applied Biosystems, Foster City, California). For qPCR, 3 μL cDNA was loaded in duplicate with TaqMan primer probes and TaqMan Master Mix (Applied Biosystems, Foster City, California) and reactions were carried out on a StepOnePlus (Applied Biosystems) for 40 cycles. In each sample, the *C*_*t*_ value of each of eight genes (*BARX1, BARX2, FBN2, GAPDH, ITM2B, LATS1, LEF1, MMP13*) was normalized to the average of the *C*_*t*_ measurements of two endogenous normalizer genes (*ATE1* and *SHOC2*; selected based on their observed stable expression across mRNA-seq samples) from the same sample [31]. The TaqMan assay product numbers (Applied Biosystems) used were as shown in Table 1. For *LATS1*, a custom TaqMan assay was used based on a 166-mer submitted sequence (provided in Additional file 2: Supplementary Note S1).

**Table 1:** qPCR validation of differential expression (sarcoma vs. skin) of five genes detected as differentially expressed by mRNA-seq. Abbreviations are as follows: n.s., not significant at *p* ≤ 0.05; Δ*C*_*t*_, average of the two differences between the cycle time for the indicated gene and the average of the cycle times for two endogenous normalizer genes (*ATE1* and *SHOC2*); ΔΔ*C*_*t*_,difference between the average Δ*C*_*t*_ value for the sarcoma samples and the average Δ*C*_*t*_ value for the normal skin samples. Column "Gene" contains the HGNC official gene symbol

Gene	RNA-seq log_2_(sarcoma/skin)	TaqMan assay	ΔΔ*C*_*t*_	qPCR log_2_(sarcoma/skin)
*BARX1*	5.29	Hs01026253_g1	436.6	8.77
*BARX2*	−7.08	Hs01573160_m1	0.0003	−11.76
*FBN2*	4.99	Cf02646808_mH	83.67	6.39
*GAPDH*	−3.86	Cf04419463_gH	0.5663	−0.82
*ITM2B*	−3.31	Hs00222753_m1	0.0303	−1.72
*LATS1*	0.815	custom (see Methods)	0.8697	−0.20
*LEF1*	−2.17	Hs00212390_m1	0.0689	−3.86
*MMP13*	5.91	Cf02741638_m1	84.62	6.40
*ATE1*	n.s.	Cf02638583_m1	n/a (endogenous normalizers)	
*SHOC2*	n.s.	Cf02707128_m1		

### Protein analysis

Total proteins were isolated from cultured cells in radioimmunoprecipitation assay (RIPA) buffer containing protease and phosphatase inhibitors (EZBlock, BioVision, Milpitas, CA). A 27 gauge needle was used to homogenize cells and cellular debris was removed with two 12,000 g centrifugations for 10 min each at 4 °C. Ten micrograms total protein, diluted in Laemmli sample buffer containing β-mercaptoethanol, were loaded onto 7.5% (for FN1) or 12% (for BARX1) Mini-PROTEAN® TGX™ acrylamide gels (BioRad Laboratories, USA) and separated by electrophoresis in a tris-glycine buffer system. Proteins were transferred to 0.2 μm nitrocellulose (BioRad), stained with Pierce™ Reversible Protein Stain Kit (Thermo Scientific, Rockford, IL), and imaged. The membranes were de-stained, rinsed in TBST, and blocked with 5% bovine serum albumin in tris-buffered saline+Tween 20 (TBST) for 1 hour at room temperature. Anti-Fibronectin (Abcam, ab6328) and Anti-Barx1 (Abcam, ab26156) were diluted at 1:2000 and 1:1000 respectively in blocking buffer, and membranes were incubated overnight at 4 °C. Following TBST rinses, secondary staining with 1:10,000 Goat Anti-Mouse-HRP (Abcam, ab6789 for FN1) and 1:40,000 Goat Anti-rabbit-HRP (Abcam, ab205718, for BARX1) occurred at room temperature for 45 min followed by rinsing in TBST, and detection with ECL Select™ Western Blotting Detection Reagent (Amersham™ #RPN2235, Italy). Image Quant LAS 4000 software was used to acquire the chemiluminescent images and digitization was performed with Image Quant TL.

### Genomic block-based test for potential somatic copy number alterations (SCNAs)

We tested for concordant up- or down-regulation (in FISS vs. skin) of mRNA levels for genes within 10 Mbp regions of the genome as a proxy measure for detecting genomic DNA copy number alterations. We aggregated gene-level RNA-seq count data sets into adjacent, non-overlapping 10 Mbp windows, genome-wide. We averaged the gene-level log_2_(sarcoma/normal tissue) ratios of the genes within each window and then used a genome-wide permutation test (1000 independent shufflings of the genes’ window assignments), rejecting the null hypothesis if the empirical *p* value (computed by comparing the window-average based on the unshuffled assignments to the sorted vector of window-averages based on the shuffled assignments) satisfied *p* < 0.001. We further filtered windows to call a “possible SCNA” only if the window-level, absolute average log_2_(sarcoma/normal tissue) value exceeded 1.0. We mapped between these window regions and previously reported recurrent FISS SCNAs [4] using chromosome coordinate overlap.

### Statistical methods

We carried out all statistical tests using the R statistical computing environment (version 3.3.3) and using R software packages from the Bioconductor system [32] release 3.4 (with the exception of gene functional enrichment analysis; see above sections “Gene Set Enrichment Analysis” and “Enrichr Analysis”). Statistical *p*-values were adjusted for multiple hypothesis tests using the false discovery rate (FDR) method [27].

### Human soft tissue sarcoma datasets

A mixture of microarray and mRNA-seq transcriptome datasets derived from human soft-tissue sarcoma and normal skeletal muscle were obtained from NCBI GEO [33] via accession numbers GSE54734, GSE6481, GSE2719, GSE2361, GSE2719, GSE7905, and GSE2193. Gene-level expression data were normalized on each sample (by mean expression level) and then merged. Each gene’s log expression levels across the samples from the merged datasets were tested for a difference of means assuming homoscedasticity (Student’s *t-*test) or heteroscedasticity (Welch’s *t-*test) as indicated by an *F-*test. For each gene for which the equal-means null was rejected at FDR ≤ 0.05), the effect size was computed as average(tumor)/average(normal).

### Canine soft tissue sarcoma mRNA-seq profiling

100 ng – 50 μg total RNA from tissue-derived RNA samples were rRNA-depleted using Dynabeads mRNA DIRECT Micro Kit; mRNA-seq libraries were prepared using Ambion RNA-Seq library construction technologies; and barcoded libraries were sequenced on an Ion Proton System at 2 × 100 bp and then aligned to the CanFam3 genome assembly using StrandNGS (Strand Life Sciences Pvt. Ltd., Bengaluru, India) and quantified and normalized using the DEseq algorithm.

### Three-species (feline, canine, and human) permutation analysis of soft-tissue sarcoma transcriptomes

For the three-species analysis of the sarcoma transcriptome, we obtained species-specific, primary tissue differential gene expression values (log_2_(sarcoma/normal)) in three studies: feline (FISS and normal skin), human (soft tissue sarcoma and normal skeletal muscle), and canine (soft tissue sarcoma and normal skeletal muscle). For the 138 genes that were differentially expressed in sarcoma vs. normal tissue in all three species [consisting of 53 genes that are upregulated in sarcoma vs. normal in all three species; 38 genes that are downregulated in sarcoma vs. normal in all three species; and 47 genes that have mixed differential expression], we constructed a 138 × 3 binary matrix (138 genes by three species) based on the differential expression values. The binary matrix contained 1, if the row’s gene is upregulated in sarcoma vs. normal tissue in FISS in the particular column’s species, and 0 otherwise. We then tested the number of rows containing (1,1,1) in each of 100,000 permuted matrices using the R function sample. We computed an empirical *p-*value as the number of times the count of (1,1,1) rows in a permuted matrix exceeded 53. We performed an identical permutation analysis for downregulated genes, assigning 1 to an entry in the binary matrix if the gene corresponding to the row is downregulated in the species corresponding to the column, and using 100,000 permutations and using 38 as the reference value.

### Other genomic datasets

We identified cat-to-human synteny blocks using the VISTA Comparative Genomics web tool [34] via the Ensembl portal (synteny analysis from Ensembl release 87). For the circos plot, we obtained cytogenetic band data for the cat genome from the UCSC genome browser portal [35], and used the cytogenetic band data for the human genome that was included with the RCircos software package [36]. We mapped cat genes to human orthologs (and vice-versa), obtained cat gene annotations, and obtained mapped Ensembl gene identifiers to official gene symbols using Ensembl Release 87. We obtained human sarcoma SCNA genes information from the cBioPortal database [37]. For the cross-species analysis of sarcoma SCNAs, we downloaded from cBioPortal a table of cytogenetic band-level amplification/deletion significance test results that were derived from a SCNA study of adult soft tissue sarcomas that was carried out by the Cancer Genome Atlas (TCGA) consortium [9]. The cBioPortal results table was based on analysis of TCGA-published gene-level SCNA data from 265 tumor samples, comprising 58 dedifferentiated liposarcomas, two desmoid/aggressive fibromatosis, 105 leiomyosarcomas, 11 malignant peripheral nerve sheath tumors, 25 myxofibrosarcomas, two pleomorphic liposarcomas, one not otherwise specified, four soft tissue sarcomas, ten synovial sarcomas, and 49 undifferentiated pleomorphic sarcoma/malignant fibrous histiocytoma/high-grade spindle cell sarcomas.

### IC50 assay

Cells were plated at 10,000/well in a 96-well plate containing DMEM supplemented with 10% FBS and pen/strep and incubated for 24 h at 37 °C and 5% CO_2_. Media with GSK-1059615 (#11569, Cayman Laboratories, Ann Arbor MI) at 60, 30, 15, 7.5, 3.75, 1.88 μM was added to triplicate wells. Control cells were exposed to the vehicle reagent (dimethyl sulfoxide, DMSO) at concentrations equivalent to the DMSO concentrations in the corresponding GSK-1059615 samples’ media. After 24 h, media was replaced with new GSK-1059615 or control media. At 48 h, media was removed and 0.004% resazurin (Acros Organics #189900010, NJ) in phosphate-buffered saline (PBS) was added. The plate was incubated for 1 h at 37 °C and 5% CO_2_. Fluorescence was measured at 530 nm excitation, 590 nm emission with a Tecan Infinite F200. Relative Fluorescence Units (RFU) were used to calculate percent of baseline metabolic activity. At each concentration, measurements were acquired in three biological replicates.

### Data release

All cat mRNA-seq data files for this study have been deposited in the National Center for Biotechnology Information (NCBI) Sequence Read Archive (SRA) [38] (see Declarations; Availability of Data and Material).

## Results

### Differential expression in feline injection site-sarcoma (FISS) vs. normal tissue

**Fig. 1:**
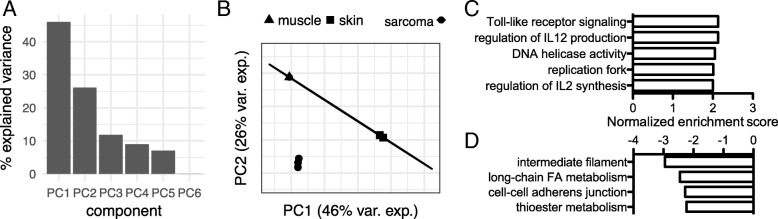
Principal components analysis (PCA) and gene functional annotation enrichment analysis of the transcriptomes of FISS and normal tissue. **a** Bars indicate the percentage of the variance of gene expression level data across the six tissue samples that is explained by each of the six principal components (PCs). Over 70% of the variance is explained by the combination of principal components 1 and 2 (PC1 and PC2). **b** PCA loading scores for each of the samples, for PC1 and PC2. Each mark corresponds to a sample, with mark shape indicating sample tissue type (see legend). Ellipses indicate groupings of normal tissue and sarcoma tumor samples. The normal skin tissue ellipse is highly eccentric due to separation between muscle and skin tissues. The sarcoma sample group ellipse is not visible due to the tight clustering of sarcoma samples in the loading plot. **c** Significant positive functional annotation enrichment scores based on Gene Set Enrichment Analysis (GSEA) of gene-level differential expression log_2_(sarcoma/skin) values, using ortholog mapping and human gene functional annotations from the Gene Ontology. Bar magnitude indicates the annotation’s normalized enrichment score from GSEA; each bar significantly differs from zero score with FDR < 0.05. Each positive bar value corresponds to a significant enrichment of genes with the indicated annotation, with positive log_2_(sarcoma/skin) values, i.e., evidence for upregulation of genes with the indicated function in sarcoma vs. normal tissue. **d** Significant negative functional annotation enrichment scores based on GSEA of gene-level log_2_(sarcoma/skin) values. Each negative bar value corresponds to a significant enrichment of genes with the indicated annotation, with negative log_2_(sarcoma/skin) values, i.e., evidence for downregulation of genes with the indicated function in sarcoma vs. normal tissue

In order to study transcriptome differences between FISS and normal tissue, we first compared the transcriptomes of FISS tumor tissue, normal skin, and normal skeletal muscle (with muscle included as an outgroup for the normal tissue samples; *N* = 6 samples total). In an unsupervised analysis (principal components analysis, PCA) of the 6252 genes with significant variance (see Methods), the muscle and skin samples were easily separable from the FISS tumor samples by principal components 1 and 2 (PC1 and PC2) (Fig. 1a, b), which together explained the majority (72%) of the total variance of gene expression. The skin samples and the FISS samples were each tightly grouped in the PCA analysis, and given the fibrosarcoma histotypes of the FISS tumors in this study, for the subsequent supervised analysis of differential expression, we compared FISS to normal skin tissue only, identifying 3049 differentially expressed genes (*q* < 0.05). In order to identify molecular functions and biological processes that are associated with the sets of genes that were up- and down-regulated in FISS vs. skin, we ranked genes by their log_2_(sarcoma/skin) values, mapped genes to human orthologs, and then analyzed sets of human genes that are annotated with specific functional ontology terms (Gene Ontology terms) [39] for enrichment among up- or down-regulated genes using Gene Set Enrichment Analysis (GSEA) [29]. For genes that are upregulated in FISS vs. skin, we found enrichment (*q* < 0.05 and absolute GSEA enrichment score > 2.0) of gene sets for five categories of gene annotation terms (Fig. 1c): Toll-like receptor signaling, regulation of interleukin 12 (IL-12) production, DNA helicase activity, replication fork, and regulation of interleukin 2 (IL-2) synthesis. For genes that are downregulated in FISS vs. skin, we found enrichment of downregulated genes in four categories of gene sets (Fig. 1d): intermediate filament, long-chain fatty acid metabolism, cell-cell adherens junction, and thioester metabolism.

### Differential expression in FISS-derived cell lines vs. skin-derived fibroblasts

**Fig. 2:**
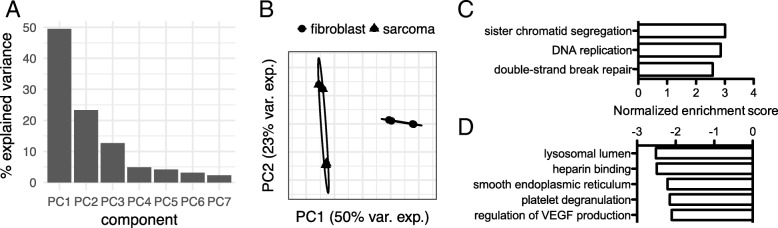
Principal components analysis (PCA) and gene functional annotation enrichment analysis of the transcriptomes of FISS-derived and skin-derived fibroblast cell cultures. **a** Bars indicate the percentage of the variance of gene expression level data, across the eight cell culture samples, that is explained by each of the first seven principal components (PCs). Over 70% of the variance is explained, collectively, by principal component 1 (PC1) and principal component 2 (PC2). **b** PCA loading scores for each of the samples, for PC1 and PC2. Each mark corresponds to a sample, with mark shape indicating the cell type (see legend). Ellipses indicate groupings of fibroblast and sarcoma-derived cells. The ellipse for the fibroblast samples is highly eccentric due to the loading plot marks being nearly collinear within the sample group. **c** Significant positive functional annotation enrichment scores based on Gene Set Enrichment Analysis (GSEA) of gene-level differential expression log_2_(sarcoma cell / fibroblast) values (obtained from cultured cells), using ortholog mapping and human gene functional annotations from the Gene Ontology. Bar magnitude indicates the GSEA normalized enrichment score; each bar significantly differs from zero score with FDR < 0.05. Each positive bar value corresponds to a significant enrichment of genes with the indicated annotation, with positive log_2_(sarcoma cell / fibroblast) values, i.e., evidence for upregulation of genes with the indicated function in sarcoma-derived vs. fibroblast-derived cells. **d** Significant negative functional annotation enrichment scores based on GSEA of gene-level log_2_(sarcoma cell / fibroblast) values. Each negative bar value corresponds to a significant enrichment of genes with the indicated annotation, with negative log_2_(sarcoma cell / fibroblast) values, i.e., evidence for downregulation of genes with the indicated function in sarcoma-derived cultured cells vs. skin-derived fibroblasts

As a complement to direct mRNA-seq of FISS tumor and normal tissue samples, we compared the mRNA transcriptomes of *cultured* FISS tumor-derived cells and skin-derived fibroblasts (two FISS-derived biological replicates and two fibroblast biological replicates each from different cats; *N =* 8 samples total; see Methods). We identified 3553 genes that were differentially expressed between the FISS-derived cell line and fibroblast sample groups (*q* < 0.05). By PCA analysis (Fig. 2a, b), the fibroblast samples were more tightly grouped than the FISS-derived samples, with PC1 and PC2 together explaining 73% of the variance in gene expression. Using GSEA, we analyzed genes ranked by their log_2_(sarcoma cell / fibroblast) expression values in cultured cells, detecting enrichment (*q* < 0.05, absolute GSEA enrichment score > 2.0) of upregulated genes in three categories of annotation gene sets (Fig. 2c): sister chromatid segregation, DNA replication (consistent with findings from the primary tissue analysis), and double-strand break repair. Additionally, we found enrichment of downregulated genes in five annotation gene sets (Fig. 2d): lysosomal lumen, heparin binding, smooth endoplasmic reticulum, platelet degranulation, and regulation of vascular endothelial growth factor (VEGF) production. For all 21,890 cat genes profiled, the mRNA-seq count data, gene annotation information, and differential expression test results for both the primary tissue analysis and the cultured cells analysis are provided online (Additional file 3: Table S2).

**Fig. 3:**
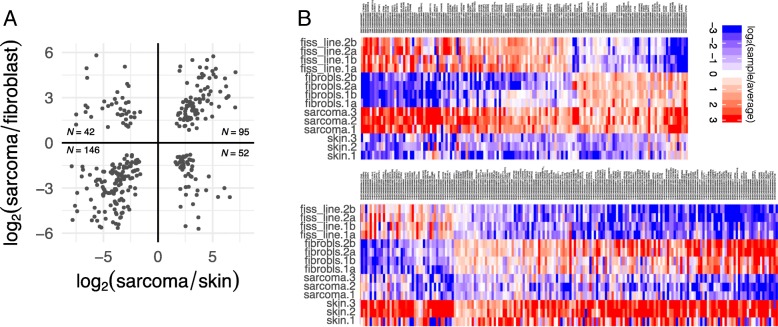
The pattern of differential expression in FISS tumors vs. normal skin tissue is consistent with the pattern of differential expression in FISS-derived cells vs. fibroblast-derived cells. **a** Each mark represents one of 335 genes that are all differentially expressed (FDR < 0.05) between the sarcoma and normal sample groups, in tissues (i.e., the comparison of sarcoma tumors vs. skin samples) and in cultured cells (i.e., the comparison of FISS-derived cell lines vs. skin-derived fibroblasts). Each data mark’s position on the horizontal axis corresponds to the log_2_(sarcoma/skin) value for the gene in tissue, and on the vertical axis corresponds to the log_2_(sarcoma cell / fibroblast) value for the gene in cultured cells. Overall counts of the number of genes within each quadrant are shown. **b** Heatmap visualization including individual gene names, with cell colors indicating the log_2_(individual sample / average across all samples) for each of 335 genes (columns) across 14 samples (rows). Red indicates upregulation vs. the sample average, and blue indicates downregulation vs. the sample average (no change from sample average is mapped to white). Genes are ordered by sign of primary tissue log_2_(sarcoma/skin) ratio, then by sign of log_2_(sarcoma cell / fibroblast) ratio in cultured cell samples, and then overall average log_2_(sarcoma/normal) ratio. Abbreviations: “fiss_line”, FISS-derived cell line; “fibrobls.”, fibroblast cells; “sarcoma”: FISS tumor tissue; “skin”: normal skin tissue

To assess the extent to which the sarcoma cell lines preserve significant molecular characteristics of FISS tumors, we investigated the degree of concordance between the set of genes that are differentially expressed between FISS tumors and skin tissue and the set of genes that are differentially expressed between the FISS-derived cell lines and fibroblasts (Fig. 3). We found 335 genes that were differentially expressed in both the tissue mRNA-seq analysis and the cell culture mRNA-seq analysis, representing a significant overlap (*p* < 10^− 16^; Fisher’s exact test; odds ratio 0.60, 95% c.i. 0.53–0.68). Moreover, among the common set of 335 “FISS marker” genes, the degree of concordance in the *direction* of the differential expression (up in both, or down in both, or up in one analysis and down in the other) was high (Fig. 3a), with an odds ratio of 6.3 (95% c.i. 3.8–10.6), and significantly differs from 1.0 at *p* < 10^− 14^ (Fisher’s Exact Test on 2 × 2 contingency table).

### qPCR validation

Based on magnitude of effect size, consistency of up- or down-regulation in sarcomas in multiple species (see Sec. Gene-level cross-species transcriptome analysis), and/or annotated gene functions suggesting regulatory importance, we selected—from the 241 genes that are consistently (with the same direction of cancer/normal fold-change in both primary tissue and in cultured cells) differentially expressed in FISS vs. normal cells—four FISS-upregulated genes (*BARX1, FBN2, MMP13*, and *LATS1*) and four FISS-downregulated genes (*BARX2, LEF1*, *GAPDH*, and *ITM2B*) for targeted validation by quantitative polymerase chain reaction (qPCR). The qPCR measurements were consistent with the mRNA-seq measurements, in terms of the direction and magnitude of the fold-change (sarcoma vs. normal) for seven out of the eight genes tested (Table 1 and Additional file 4: Figure S1; *r* ^2^ = 0.879).

### Cross-species TSG/oncogene analysis

**Fig. 4:**
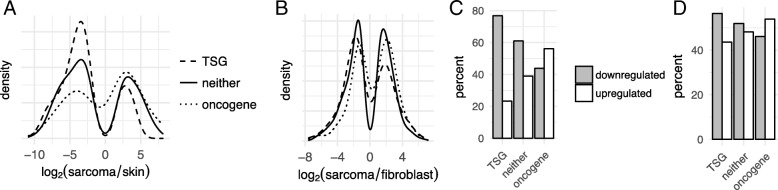
Patterns of differential expression (sarcoma vs. normal) of feline orthologs of human oncogenes and tumor suppressor genes (TSGs) significantly differ from those of feline genes whose orthologs are not oncogenes or TSGs. **a** Estimated density distributions of log_2_(sarcoma/skin) values for 1542 feline genes that are differentially expressed in tissues and that are orthologs of human TSGs, oncogenes, or genes that are neither TSGs nor oncogenes (“neither”). **b** Estimated density distributions of log_2_(sarcoma cells / fibroblast) values for 3049 feline genes that are differentially expressed in cultured cells and that are orthologs of human TSGs, oncogenes, or genes that are neither TSGs nor oncogenes (“neither”).**c** Proportions of genes that are up- or down-regulated in sarcoma vs. normal skin tissue, for 1542 feline genes classified by their human orthologs’ annotations (“TSG”, “oncogene”, or “neither”) show a clear trend of increasing odds of upregulation, between TSG, neither, and oncogene groups. **d** Proportions of genes that are up- or down-regulated in sarcoma-derived cells vs. skin-derived fibroblasts, for 3049 feline genes classified by their human orthologs’ annotations (“TSG”, “oncogene”, or “neither”) show a clear trend of increasing odds of upregulation, between TSG, neither, and oncogene groups

Insofar as little is known about the genes whose dysregulation in FISS presumably drives disease progression, we tested whether cat orthologs of (i) known human oncogenes, (ii) known human tumor suppressor genes (TSGs), or (iii) human genes that are neither oncogenes nor TSGs have different distributions of log_2_(sarcoma/normal) expression values. Based on a similar analysis of cancer transcriptome data in our previous study of canine bladder cancer [12], we hypothesized that cat orthologs of human TSGs would be more likely to be downregulated in FISS, and similarly, that cat orthologs of human oncogenes would be more likely to be upregulated in FISS. Indeed, kernel density analysis of the log_2_(sarcoma/skin) values for the 1542 cat genes that we had found to be differentially expressed in FISS tumor vs. skin, grouped by the annotation status of the human ortholog of the cat gene (TSG, oncogene, or neither^1^) were consistent with our hypothesis (Fig. 4a; Table 2). The odds ratios for a gene to be upregulated in FISS (conditioned on the annotation of its human ortholog) differ from 1.0 at *p* < 0.001 (Fisher’s Exact Test (FET) on 2 × 3 contingency table). We also tested the hypothesis for the cultured cells mRNA-seq data by plotting the kernel density-smoothed distributions of log_2_(sarcoma cell / fibroblast) values for the 3049 genes that were differentially expressed in sarcoma-derived cells vs. fibroblasts, conditioned on the human ortholog’s annotation (TSG, neither, or oncogene). In the resulting density plots (Fig. 4b) the effect of the human ortholog’s annotation on the density distribution of log ratios was not as strong but it was still evident (Table 2). In this case, the FET was not significant (*p* = 0.22) but distributions of ranks of log_2_(sarcoma cell / fibroblast) values in cultured cells differ significantly between the “TSG” and “oncogene” sets of feline genes (*p* < 0.001, Kolmogorov-Smirnov test). For both the tissue (Fig. 4c) and cultured cells (Fig. 4d) mRNA-seq datasets, the effect of the cat gene’s human ortholog’s annotation status on the proportions of genes that are up- or down-regulated in FISS is clearly evident.

**Table 2:** Among cat genes that are differentially expressed in sarcoma vs. normal skin and that have human orthologs, the odds that the gene is upregulated in sarcoma conditioned on the human ortholog's TSG/oncogene annotation. Results are shown for both the primary tissue analysis (FISS tumor vs. normal skin) and the cultured cells analysis (FISS-derived cells vs. cultured fibroblasts)

Human ortholog annotation	Odds that the cat gene is upregulated in FISS tumor vs. normal skin	Odds that the cat gene is upregulated in FISS-derived cells vs. fibroblasts
TSG	0.30	0.77
neither TSG nor oncogene	0.64	0.93
oncogene	1.28	1.17

### Somatic copy number alteration analysis

**Fig. 5:**
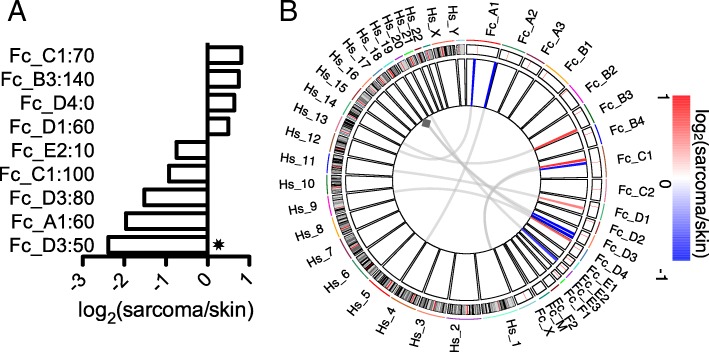
Analysis of genomic regions of coherent up- or down-regulation in sarcoma vs. normal tissue. **a** 10-Mbp genomic regions showing statistically significant (*p* < 0.05, based on permutation test, and absolute log_2_(sarcoma/skin) > 1) and coherent up- or down-regulation of the genes within the region, in sarcoma vs. normal skin tissue. Each bar corresponds to a specific 10 Mbp region in the cat genome. Bars are labeled by *Felis catus* chromosome and the start coordinate of the region, in Mbp (e.g., “Fc_C1:70”). Bars indicate the average log_2_(sarcoma/skin) values for all genes within the indicated region. Asterisk indicates a concordance of the transcriptional analysis of the indicated region with a recurrent deletion in FISS as reported in a previous array comparative genomic hybridization study [4]. **b** Circos-style graphical depiction of coherently up- (red spokes) or down- (blue spokes) regulated 10 Mbp regions in sarcoma vs. normal skin (see colormap). Cat nuclear chromosomes are arranged clockwise around the circos plot from 12:00 to 5:00; human nuclear chromosomes are arranged clockwise from 5:00 to 12:00. Curved light gray arcs indicate syntenic regions of the human genome that correspond to the regions of coherent up- or down-regulation in FISS. Gray diamond denotes syntenic human genomic region that is recurrently deleted in human soft-tissue sarcoma (see Methods), according to the cBioPortal database [37]

Recurrent somatic copy number alterations (SCNAs) have been reported in FISS and for feline soft-tissue sarcomas that are not injection-site-associated [4] and previous studies of other cancers have successfully detected known SCNAs through region-based analysis of tumor mRNA-seq data [40]. Thus, we analyzed the gene-level differential expression data for FISS vs. skin samples to identify large chromosomal regions of coherent up- or down-regulation at the transcript level. We divided the cat genome into 10 Mbp windows and within each window, we compared the average of the log_2_(sarcoma/skin) values [denoted by angle brackets, ⟨log_2_(sarcoma/skin)⟩] for all the genes in the window to a background distribution (see Methods). This permutation analysis yielded a *p-*value for each window, and we found nine putative SCNA regions for which *p* < 0.001 and | ⟨log_2_(sarcoma/skin)⟩| > 1.0. Of these regions, four show coherent upregulation (in sarcoma vs. normal skin) of the genes within them and five show coherent downregulation (Fig. 5a). Of these regions, the region (50–60 Mbp on chromosome *Fc*-D3) that was the most downregulated transcriptionally in our study was previously reported to have a recurrent somatic deletion in FISS [4].

### Cross-species SCNA analysis

Hypothesizing that SCNAs that drive cancer progression in one species may correspond to SCNAs in the syntenic genomic region for a related cancer in another species, we mapped putative SCNA regions of the cat genome to their syntenic regions in the human genome and then tested whether the human regions have recurrent SCNAs in human soft tissue sarcomas. We found a feline genomic region harboring an SCNA in FISS whose human syntenic region was previously reported to have a recurrent SCNA in soft-tissue sarcoma [9] (Fig. 5b). The 50–60 Mbp region of *Fc*-D3 coincides with a previously reported recurrent chromosomal deletion in FISS and in non-injection-site-associated feline sarcoma [4]; its human syntenic region spans 20.9–80.3 Mbp on *Hs*-18 (*Hs*-18q23), which is recurrently deleted in human soft-tissue sarcoma (*q* < 0.02 based on analysis of SCNA data from ref. [9] for the 13 genes in *Hs*-18q23). Detailed information on the nine putative FISS SCNAs is provided online (Additional file 5: Table S3).

### Gene-level cross-species transcriptome analysis

**Fig. 6:**
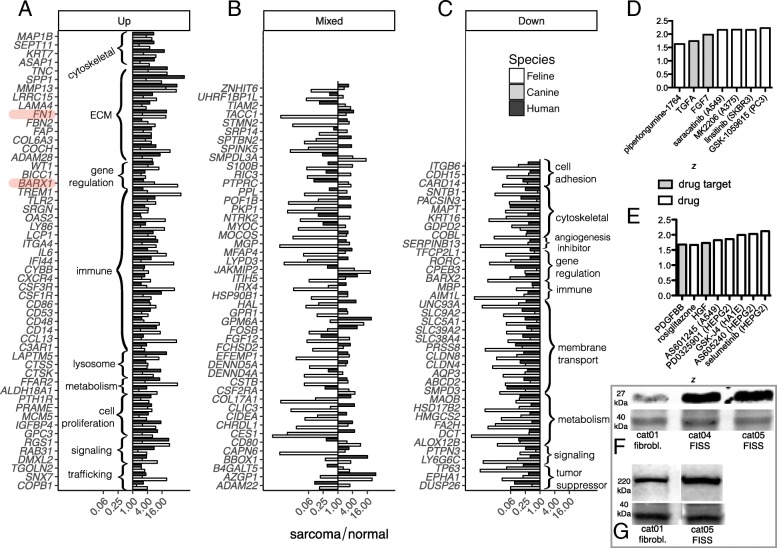
Cross-species (feline, canine, and human) analysis of soft-tissue sarcoma transcriptomes shows significant overlap. **a** Species-specific differential expression ratios (sarcoma/normal) of 53 genes that were selected because they are upregulated in sarcoma vs. normal tissue in three species: FISS, human soft tissue sarcoma, and canine soft tissue sarcoma. Genes are grouped vertically by gene annotation (curly braces). ECM, extracellular matrix. Gene symbol mappings to stable gene identifiers are provided in Additional file 3: Table S2 online. Gene symbols highlighted in red indicate genes whose upregulation in FISS was validated at the protein level. **b** Species-specific differential expression ratios (sarcoma/normal) of 47 genes that have mixed differential expression (i.e., not all upregulated and not all downregulated) among the feline, canine, and human sarcoma transcriptome profiling studies. **c** Species-specific differential expression ratios (sarcoma/normal) of 38 genes that are downregulated in sarcoma vs. normal tissue in FISS, human soft tissue sarcoma, and canine soft tissue sarcoma. Genes are grouped vertically by gene annotation (curly braces). **d** Cell-line-transcriptome-based bioinformatic screen for drug and drug-target enrichment among 53 genes that are upregulated in sarcoma in all three species. White bars correspond to pairs of drugs and cell lines (or drugs only, representing results from a screen against multiple cell lines), and gray bars correspond to drug targets. Bar height corresponds to the enrichment *z* score from Enrichr. Cell lines are indicated parenthetically. **e** Cell-line-transcriptome-based bioinformatic screen for drug and drug-target enrichment among 38 genes that are downregulated in sarcoma in all three species. Bar height indicates the Enrichr *z* score and bar fill color indicates whether the enrichment is for a drug or drug-target, as in panel (**d**). **f** Membrane image of immunoblot for protein BARX1 in lysates from fibroblasts (“cat01 fibrobl.”) and FISS-derived cell lines (“cat04 FISS cells” and “cat05 FISS cells”). The 27 kDa chain is BARX1 and the 40 kDa chain is a normalization control band from Memcode total protein stain (see Methods). Full membrane image is available as Additional file 7: Figure S2A–B. Quantified band intensities are provided in Additional file 6: Table S4. **g** Membrane image of immunoblot for protein FN1 in lysates from fibroblasts (“cat01 fibrobl.”) and FISS-derived cell lines (“cat05 FISS cells”). The 220 kDa chain is FN1 and the 40 kDa chain is a normalization control band from Memcode total protein stain (see Methods). Full membrane image is available as Additional file 7: Figure S2C–D. Quantified band intensities are provided in Additional file 8: Table S5

Next, we investigated the extent of overlap of the FISS transcriptome with the transcriptomes of human and dog soft-tissue sarcomas. To do so, we obtained differential gene expression (log_2_(sarcoma/normal)) values for a human study (a published transcriptome profiling study of soft sarcoma and normal skeletal muscle tissues; see Methods) and a canine study (mRNA-seq profiling of banked soft tissue sarcoma and normal skeletal muscle samples; see Methods). Via ortholog mapping, we obtained log_2_(sarcoma/normal) values for each orthologous gene triple (cat/dog/human) across all three species. We found 138 genes that were differentially expressed in sarcoma vs. normal tissue in all three species: 53 genes that are upregulated in sarcoma vs. normal in all three species (Fig. 6a); 38 genes that are downregulated in sarcoma vs. normal in all three species (Fig. 6c); and 47 genes that have mixed differential expression in the three species (Fig. 6b). Using a permutation approach, we determined that the observed degree of concordance (91 genes out of 138 having consistent fold-change signs in all three species) was extremely unlikely to occur by chance (by permutation test, *p* < 10^− 5^ for the upregulated genes and *p* < 10^− 5^ for the downregulated genes; see Methods).For the 47 genes with inconsistent directions of differential expression across the three species, the FISS and canine soft-tissue sarcoma had more ortholog pairs with consistent fold-change directions (19 ortholog pairs) than FISS and human soft-tissue sarcoma (13 ortholog pairs) and canine and human soft-tissue sarcoma (15 ortholog pairs).

### In silico screen for potential new treatment approaches FISS

Results from previous cancer drug repositioning studies have supported the idea that drugs whose transcriptional effects in human cell lines are substantially opposite (in terms of treatment-vs.-vehicle fold changes, across genes that are transcriptionally altered by the drug treatment) the transcriptional alterations that are measured in tumor vs. normal tissue are more likely to have cancer-inhibiting effects [41, 42]. Thus, we screened the sets of 53 and 38 genes that are consistently dysregulated (upregulated and downregulated, respectively) in soft-tissue sarcoma in cats, dogs, and humans (Fig. 6a, c) against ten previously published drug-to-cell-line response datasets [43] using the web tool Enrichr [30] in order to identify drugs and drug targets that may be therapeutically beneficial for sarcoma treatment. For the sarcoma-upregulated genes, the screen identified (FDR < 0.05) five drugs and two drug targets (Fig. 6d; Table 3), and for the sarcoma-downregulated genes, the screen identified six drugs and two drug target protein families (Fig. 6e; Table 3).

**Table 3:** Functional information for drugs and drug targets identified by bioinformatic screening of genes that are consistently upregulated or downregulated in soft tissue sarcomas, across three species. Screening of sets of 53 genes that are upregulated in sarcoma and 38 genes that are downregulated was performed using the Enrichr web tool (see Methods and Fig. 6)

gene set	drug or target	comment	reference
up in sarcoma	piperlongumine-1764	natural product with anti-cancer properties	[44]
up in sarcoma	TGFA (transforming growth factor ɑ)	targeted for immunotoxin therapy	[45]
up in sarcoma	FGF7 (fibroblast growth factor 7)	proposed therapeutic target	[46]
up in sarcoma	saracatinib (AZD-0530)	investigational anti-cancer drug; dual-kinase inhibitor (Src and Bcr-Abl)	[47]
up in sarcoma	MK-2206	investigational anti-cancer drug; selective protein kinase B (Akt) inhibitor	[48]
up in sarcoma	linsitinib (OSI-906)	investigational anti-cancer drug; inhibitor of insulin receptor and insulin-like growth factor 1 receptor	[49]
up in sarcoma	GSK-1059615	phosphatidylinositol 3-kinase inhibitor and mTOR inhibitor	[50]
down in sarcoma	PDGFBB (platelet-derived growth factor BB monomer)	frequently drives sarcoma growth via autocrine signaling	[51]
down in sarcoma	rosiglitazone	peroxisome proliferator-activated receptor-ɣ agonist	[52]
down in sarcoma	HGF (hepatocyte growth factor)	HGF-MET signaling axis implicated in soft tissue sarcoma proliferation	[53]
down in sarcoma	AS601245	c-Jun N-terminal kinase inhibitor; proposed as a co-therapeutic with rosiglitazone	[54]
down in sarcoma	PD0325901	mitogen-activated protein kinase kinase (MAPK/ERK kinase or MEK) inhibitor	[55]
down in sarcoma	GSK-J4	prodrug of GSK J1, a selective inhibitor of H3K27 histone demethylases JMJD3 and UTX	[56]
down in sarcoma	AS605240	phosphatidylinositol 3-kinase inhibitor	[57]
down in sarcoma	selumetnib (AZD-6244, ARRY-142886)	ATP-independent inhibitor of mitogen-activated protein kinase kinase (MEK or MAPK/ERK kinase) 1 and 2; investigational cancer drug	[58]

### Immunoblot analysis of BARX1 and FN1 expression in FISS

Among the 53 genes that are transcriptionally upregulated in FISS as well as in soft-tissue sarcomas (vs. normal tissue) in dogs and humans, we validated at the protein level the upregulation of two genes (BARX1, BarH-like homeobox 1; and FN1, fibronectin 1) in FISS-derived cells vs. skin-derived fibroblasts. By immunoblot with quantitative image analysis we measured that BARX1 protein is three-fold more abundant in FISS-derived cells than in skin-derived fibroblasts (Fig. 6f, Additional file 6: Table S4, Additional file 7: Figure S2), and that FN1 protein is 2.1-fold more abundant in a FISS-derived cell line (cat05) than in skin-derived fibroblasts (Fig. 6g, Additional file 8: Table S5, Additional file 7: Figure S2), though FN1 was not consistently upregulated at the protein level in all FISS-derived cell lines (Additional file 7: Figure S2).

### IC50 assay of GSK-1059615

**Fig. 7:**
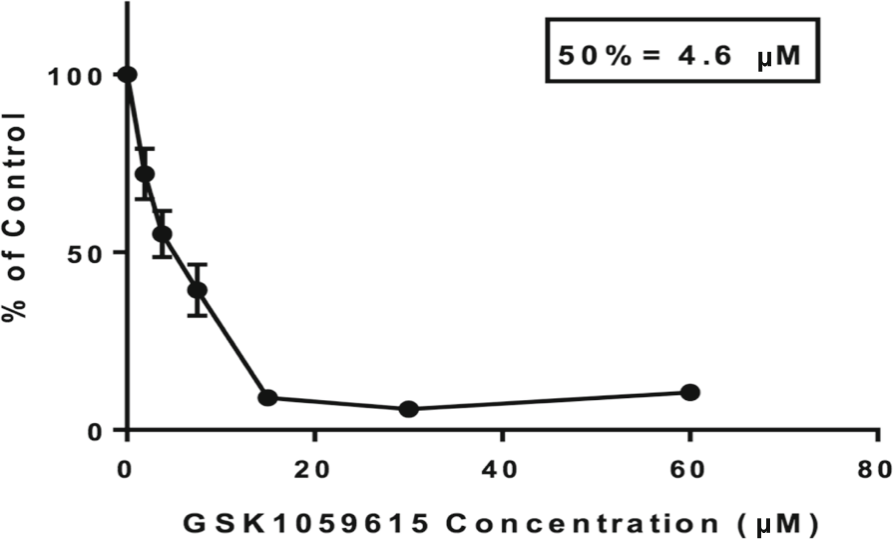
Compound GSK-1059615 potently inhibits growth of FISS cells in vitro*.* A 50% inhibitory concentration (IC50) assay analysis of cells derived from the FISS tumor of cat04 (see Methods) incubated for 48 h with GSK-1059615 indicates that the compound inhibits FISS-derived cell growth in vitro at micromolar concentrations. Marks indicate average resazurin fluorescence of cells in three biological replicates (bars, sample standard deviation) at the indicated concentration of GSK-1059615, in each case as a % of the fluorescence intensity of control cells incubated with identical vehicle concentration (see Methods)

>Because the compound GSK-1059615 (an inhibitor of phosphoinositide 3-kinases (PI3K) and mammalian target of rapamycin (mTOR)) had the strongest enrichment in the in silico screen (Fig. 6d) and based on its molecular targets as described in Discussion, we selected it for an in vitro growth inhibition assay. We found that 48 h incubation with GSK-1059615 potently inhibited growth of FISS-derived cells in vitro, with an IC50 of 4.6 μM (Fig. 7).

## Discussion

This work was principally motivated by three questions: (1) What genes are differentially expressed in FISS, and what are their biological pathways and functions? (2) In which genomic regions are genes coherently differentially expressed in FISS vs. normal tissue? and (3) To what extent does the FISS transcriptome overlap with those of dog and human sarcomas?

The 3049 genes that we detected with altered transcript abundances in FISS tumors vs. normal skin comprise genes in multiple pathways that could plausibly play a role in the pathogenesis or progression of FISS.

### Sarcoma-upregulated genes that are common to sarcomas in the three species

Among the genes that are upregulated in sarcoma in all three species, several genes stand out: *FAP* (fibroblast activation protein α) is known to be expressed in soft tissue sarcomas and is involved in control of fibroblast growth [59], *WT1* (Wilms tumor suppressor) is expressed in over half of soft tissue sarcomas and has prognostic significance [60], and *PRAME* (preferentially expressed antigen in melanoma) promotes cell growth, is expressed in tumors including soft tissue sarcomas, and has been proposed as a potential immunotherapy target for sarcoma [61]. Additionally, several enriched gene annotations are notable. Many of the upregulated genes are associated with the immune system or complement system (*TLR2*, *CD14*, *C3AR1*, *ITGA4, CXCR4*, *IFI44*, *CSF3R*, *CSF1R*, *CD53*, *CD48*, *IL6*, *CCL13*, *CD86, LY86, LCP1, OAS, SRGN, CTSS, TREM1*) or the extracellular matrix (ECM) (*TNC, SPP1, MMP13, LRRC15, LAMA4, FN1, FBN2, FAP, COL6A3, COCH, ADAM28*). Other cellular functions or biological processes with which multiple consistently upregulated genes are annotated include cell proliferation (*PTH1R, PRAME, MCM5, IGFBP4, GPC3*), cytoskeleton (*MAP1B, SEPT11, KRT7, ASAP1*)*,* gene regulation (*WT1, BICC1, BARX1*), lysosome (*LAPTM5, CTSS, CTSK*), signaling (*GPC3, RGS1, RAB31*), trafficking (*TGONL2, SNX, COPB1*), and metabolism (*FFAR2, ALDH18A1*).

The finding of enrichment of ECM-related genes among the 53 genes that are upregulated in sarcoma is consistent with the abundant ECM typically seen in histopathological analysis of FISS tumors [3] and the upregulation of both ECM structural constituents (*COL6A3, FBN2, LAMA4*), as well as degradative enzymes (*MMP13, ADAM28*) is suggestive of ECM remodeling. Of note, increased expression of orthologous genes regulating ECM remodeling was also seen in human and canine sarcomas (Fig. 6a), which may suggest that some molecular mechanisms of FISS invasiveness are shared with soft-tissue sarcomas in dogs and humans.

At the level of specific cytokine pathways, we found enrichments for genes associated with two specific cytokines (interleukin-2 and interleukin-12) among the 53 genes that are upregulated in sarcoma. Genes annotated as having functions related to regulation of production of interleukin-2 (IL-2) (e.g., *CD86, CD28, PRKCQ, CD80, IL1A, LAG3, IL1B, IL17F, CD276, GLMN)* were enriched at FDR < 0.05 with a GSEA enrichment score of 2.0. Interleukin-2 is primarily produced by activated CD4+ T lymphocytes, and it promotes the differentiation of immature T cells into regulatory T cells. Lymphocytic aggregates have been reported in approximately 59% of FISS cases, and these are typically enriched for T cells [62]. IL-2 has antitumor activity and, in humans, recombinant IL-2 immunotherapy has been used clinically for treating melanoma. In the context of feline fibrosarcoma, adjuvant immunotherapy with local injection of recombinant human IL-2 extended survival times [63] and local canarypox-vectored recombinant IL-2 immunotherapy significantly reduced recurrence rates in cat fibrosarcomas [64, 65]. Genes annotated as having functions related to the regulation of production of the cytokine interleukin-12 (IL-12) (e.g., *ACP5, CCR7, MAST2, IDO1, RIPK2, TLR4, IL10, MEFV, TLR2, IRF8, NFKB1, IFNG, JAK3, TIRAP, IL12B)* were enriched among genes upregulated in FISS at FDR < 0.02 with a GSEA enrichment score of 2.2. Interleukin-12 is produced by activated phagocytic cells such as macrophages, dendritic cells, and neutrophils; it influences both innate and adaptive arms of the immune system, including augmenting cytotoxic CD8+ T-cell activation [66], reprogramming T helper 17 (T_H_17) cells into a T_H_1 phenotype, and reprogramming tumor-associated antigen-presenting cells to prime cytotoxic T-cells for antitumor activity [66, 67]. Inflammation is a hallmark of FISS, with lymphoplasmacytic infiltrates always present in the tumor as well as macrophages and neutrophils; furthermore, significant aggregates of macrophages are present in or at the periphery of approximately 25% of tumors [62]. In a mouse xenograft model of human rhabdomyosarcoma, administration of anti-histone antibody-conjugated IL-12 caused long-term remission and increased survival [68]. In cats, systemic IL-12 administration for treatment of spontaneous soft-tissue sarcoma has been evaluated in a phase-one clinical trial and achieved dose-dependent delivery of IL-12 to the tumor [69]. Overall and together with our transcriptional findings for genes related to IL-12 regulation, these results are suggestive that IL-12 immunotherapy may prove beneficial in the treatment of FISS.

### Sarcoma-downregulated genes that are common to sarcomas in the three species

Among the genes that are downregulated in sarcoma in all three species, there are three known tumor suppressors: *TP63* [70], *EPHA1* [71]*, and DUSP26* (which may alternately function as an oncogene depending on the cancer context) [72]. Many of the downregulated genes are associated with membrane transport (*UNC93A, SLC9A2, SLCL5A1, SLC39A2, SLC38A4, PRSS8, CLDN8, CLDN4, AQP3, ABCD2, SMPD3*) or metabolism (*MAOB, HSD17B2, HMGCS2, FA2H, DCT, ALOX12B*). Other cellular functions or biological processes for which multiple consistently downregulated genes are annotated include gene regulation (*TFCP2L1, RORC, CPEB3, BARX2*), signaling (*PTPN3, LY6G6C*), immune function (*MBP*, *AIM1L*), cytoskeleton (*SNTB1, PACSIN3, MAPT, KRT16, GDPD2, COBL*; see also Fig. 1d, “intermediate filament”), and cell adhesion (*ITGB6, CDH15, CARD14*; see also Fig. 1d, “cell-cell adherens junction”). The latter gene category is perhaps unsurprising given that there is altered and often reduced cell-cell adhesion and cell-extracellular matrix (cell-ECM) interaction in many cancers [73] and given the invasive nature of FISS. Among the cross-species downregulated genes, there are two additional genes that particularly noteworthy: *SERPINB13* is an angiogenesis inhibitor [74] that is downregulated in head and neck cancers [75], and the neutral sphingomyelinase *SMPD3* is a cell cycle regulator [76].

The finding that many genes with functions in fatty acid (FA) metabolism, such as multiple FA elongases (Additional file 3: Table S2), fatty acid 2-hydroxylase (*FA2H*), and arachidonate 12-lipoxygenase (*ALOX12B*, which produces an eicosanoid signaling molecule that stimulates epidermal lipid envelope synthesis; see Fig. 6c) are downregulated in sarcoma vs. normal skin (see also Fig. 1d, “long-chain fatty acid metabolism”) is interesting. Many tumors are lipogenic in order to sustain cell proliferation and tumor growth [77] and skin has a modest overall rate of FA synthesis, on a per-gram basis, compared to other normal tissues [78]. On the other hand, the finding that the angiogenesis-inhibiting gene *SERPINB13* is expressed at lower levels in soft-tissue sarcoma vs. normal tissue is not surprising (Fig. 6c), given the strong evidence for the role of the neovasculature in supporting sarcoma tumor progression [79].

Parallel examination of tissue samples and cultured cells reveals that there is an overall high degree of concordance in differential expression of genes in FISS tissue versus normal skin compared to FISS-derived cell lines versus cultured fibroblasts. Overall, this finding strengthens the utility of this in vitro model with one caveat. Our cross-species analysis of tumor suppressor gene (TSG)/oncogene expression suggests that bias in differential expression of feline orthologs to known oncogenes and TSG is not as strong in cultured cells as it is in primary tissue (Table 2); this could be a reflection of shared selective pressure imposed by environmental parameters of the cell culture systems.

Over 158 recurring focal SCNAs have been identified in a recent survey of copy number alterations in human cancers and cancer cell lines [80]. Although not all of these SCNAs contain known cancer-target genes, most occur in more than one human cancer type. Human soft tissue sarcoma is known to have many recurrent SCNAs, including deletions and amplifications, that range from focal to broad in scale [81]. Our region-based analysis of tumor mRNA-seq data for coherently up- or down-regulated genes in FISS versus skin identified nine putative recurring SCNAs. By our mRNA-seq-based method, we found one putative SCNA (a probable deletion on *Fc-*D3) that overlapped with a recurrent SCNA detected in a previous DNA-based analysis of feline sarcoma [4]. Overall, this modest amount of overlap is not unexpected given the limited size of the primary tissue dataset used in this study and the differing measurement modality. However, our finding does bolster the likely functional significance of the recurrent *Fc*-D3 deletion in FISS. One of the nine putative SCNA regions, *Fc*-C1 (70–80 Mbp) which had the highest-magnitude overall effect on transcription of any of the putative SCNA regions in FISS, is particularly noteworthy. Its human syntenic region, *Hs*-18q23, is recurrently deleted in sarcomas and contains two genes, *GALR1* and *CYB5A*, that are annotated as tumor suppressors [82].

The eight genes whose differential expression in FISS vs. normal skin we chose to validate by qPCR include three transcription factors (*BARX1* and *BARX2* [83], which encode BarH-like homeobox transcription factors 1 and 2 which are normally expressed in gastrointestinal tissue; and *LEF1*, which encodes lymphoid enhancer binding factor 1, a transcription factor downstream of the Wnt / β-catenin pathway [84]); two genes encoding extracellular proteins (*MMP13*, which encodes matrix metallopeptidase 13, which is involved in degrading extracellular matrix proteins such as fibrillar collagen and fibronectin and which has a role in controlling angiogenesis in some cancers [85]; and *FBN2*, which encodes an extracellular protein, fibrillin-2, that is a structural component of microfibrils in the extracellular matrix [86]); and a tumor suppressor (*LATS1*, which encodes large tumor suppressor kinase 1, a serine/threonine kinase that complexes with cell division cycle protein 1 (CDC1) in early mitosis [87]). Overall, we validated seven out of the eight genes originally identified as differentially expressed through mRNA-seq that were assayed by qPCR, with the exception being *LATS1* (notably, it was the only gene for which a custom hydrolysis probe-based qPCR assay had to be designed due to the absence of an off-the-shelf commercial assay). Among all eight genes, the expression ratios as measured by mRNA-seq and qPCR were highly concordant, with *r* ≠ 0 significant at *P* < 0.0001. Given that *BARX1* transcript abundance is nearly forty-fold higher in FISS than in skin, and given our interest in the FISS-upregulated gene *FN1* (a five-fold FISS-upregulated gene that encodes fibronectin-1, an ECM protein that has an important role in cell adhesion, growth, migration, and differentiation and whose expression is associated with more advanced disease in renal cancer [88]), we selected BARX1 and FN1 to assay at the protein level. The findings that the gastrointestinal transcription factor BARX1 is upregulated (three-fold) in FISS vs. normal skin and that the ECM protein FN1 is upregulated (two-fold) in *some* FISS-derived cell lines vs. normal fibroblasts are worthy of further focused investigation to determine their specific functions in the pathogenesis of FISS.

Of the fifteen drugs and drug targets that were identified through the screen of drug-to-cell-line databases using the sets of genes that are coherently upregulated or downregulated in sarcoma (across studies in three species), the compound GSK-1059615 is particularly interesting. GSK-1059615 inhibits PI3K and mTOR and it has been investigated as a potential antineoplastic in humans [89]. The finding (Fig. 7) that GSK-1059615 potently inhibits growth of FISS-derived cells suggests that dual PI3K and mTOR targeting is worthy of further study as a potential therapeutic approach for FISS. Additionally, the platelet-derived growth factor BB monomer (PDGF-BB) is noteworthy as a potential target. Members of the platelet derived growth factor family, including PDGF-BB, act as mitogens on mesenchymal cells, often in an autocrine fashion [90]. Depending on the cell type, PDGF-BB signaling can activate cell proliferation in the absence of inflammation. Tumor-derived PDGF-BB promotes pericyte recruitment and leads to increased stability of intratumoral vasculature, tumor cell proliferation, and survival. Notably, the anti-PDGF-A antibody olaratumab has shown promise in treatment of advanced human soft tissue sarcoma [91]. Together, these previous and new findings suggest that PDGF-BB inhibition may be worthy of study in the treatment of FISS. Also intriguing is the identification of the drugs AS601245, a Janus N-terminal kinase inhibitor, and rosiglitazone, a PPARɣ selective agonist, as their co-administration to colon cancer cells has been reported to synergistically reduce cell migration [54].

## Conclusions

In addition to providing a comprehensive molecular picture of FISS that will inform future targeted studies, this work has three principal conclusions. First, our results provide strong evidence that window-based analysis of mRNA-seq data can uncover somatic copy number alterations. Second, the results show that the transcriptome of FISS-derived cells is highly consistent with that of FISS tumors, bolstering the relevance of FISS-derived cultured cells as a model of FISS tumor cancer cells. Finally, the results of this study show that at the level of gene expression, FISS has a high degree of similarity to soft-tissue sarcomas in dogs and humans. While the use of systems biology approaches to map the molecular basis of feline cancers is still in its early stages, the present work illustrates the promise of integrating mRNA-seq with cross-species analytical approaches for uncovering candidate mechanisms underlying FISS pathogenesis and for uncovering new therapeutic approaches (such as dual targeting of the PI3K and mTOR pathways) for this challenging disease.

## Additional files

Additional file 1: **Table S1.** Sample information for the FISS transcriptome profiling. Each row corresponds to an RNA sample. Columns are as follows: sample name, the coded sample name used within this manuscript; biosample type, indicates whether the sample came from primary tissue or cultured cells; normal or cancer, distinguishes whether the sample is normal or cancerous; tissue type, gives the tissue subtype, such as sarcoma, skin, or muscle; Cat, the coded cat patient ID for this study; batch, the mRNA-seq batch number (see Methods); “#reads”, the total number of reads derived from the sample; “#uniquely mapped reads”, the number of reads that mapped to a unique location in the cat genome; %alignment, the percentage of the total number of reads that aligned to a unique location in the cat genome; #genes with non-zero count, the number of genes for which the count of aligned reads is greater than zero. (XLSX 11 kb)

Additional file 2: **Supplementary Note 1**: This file contains the cDNA sequence used for the qPCR assay design for the gene *LATS1*. (PDF 42 kb)

Additional file 3: **Table S2.** Differentially expressed genes in FISS. mRNA-seq gene expression data for 21,890 cat genes in all feline tissue and cell culture samples in this study. Columns as follows: *Geneid*, Ensembl identifier for the gene; *Chrom*, identifier of the cat chromosome (MT, mitochondrial chromosome; chromosome identifiers starting with “JH” are unplaced scaffolds) to which the gene has been mapped; *Gene Start*, leftmost chromosomal coordinate of the gene in the genome assembly; *Gene End*, rightmost chromosomal coordinate of the gene; *Strand*, the direction of transcription of the gene (1 denotes positive strand, − 1 denotes negative strand); *Symbol*, official gene symbol; *Description*, gene description field; Columns 8–21, absolute RNA-seq tag counts within the indicated gene (row), in the indicated sample (column); *log*_*2*_*_fold_change_tissue*, log_2_(sarcoma/skin) value (or “NA” if not statistically significantly different from zero); *p_adj_tissue*, adjusted *p-*value for expression level comparison between the sarcoma and skin sample groups; *log*_*2*_*_fold_change_cultured_cells*, log_2_(sarcoma cell / fibroblast) value (or NA if not detected as nonzero with statistical significance); *p_adj_cultured_cells*, adjusted *p-*value for expression-level comparison between the sarcoma cell line and fibroblast sample groups. (XLSX 4618 kb)

Additional file 4: **Figure S1.** Concordance of qPCR measurements with mRNA-seq measurements of relative gene expression in FISS vs. skin samples. Each mark represents a gene (genes are as described in Table 1) and marks are labeled by official HGNC gene symbol. (PDF 26 kb)

Additional file 5: **Table S3.** Regions of coherent up- or down-regulation in sarcoma vs. normal tissue. Each row corresponds to a 10 Mbp region of the cat genome for which the expression levels of the genes within the region are (together) consistently up- or down-regulated in sarcoma tumor tissue vs. normal skin. Columns as follows: *Fc chr*., cat chromosome; *Fc pos*. (Mbp), the chromosomal coordinate of the start of the region, in Mbp; *Genes within region*, the Ensembl gene identifiers of all genes that are annotated within the region; *log*_*2*_*(sarcoma/normal)*, the average of the log_2_(sarcoma/normal) expression values for all genes in the region; *Hs chr*., the chromosome of the human genome region that is syntenic to the indicated cat genome region; *Hs pos*, the coordinates (in the GRCh38 genome assembly) of the human genome region that is syntenic to the indicated cat genome region. (DOCX 128 kb)

Additional file 6: **Table S4.** Quantification of protein expression of BARX1 in fibroblasts and FISS cells. Columns as follows: “27 kDA BARX1”, integrated intensity of the BARX1 band at 27 kDa; “40 kDa Memcode”, integrated intensity of the normalization control chain at 40 kDa; “BARX1/Mem”, ratio of intensity of the band at 27 kDa to the intensity of the band at 40 kDa, for the indicated row; “FISS/fibrobl”, ratio of “BARX1/Mem” for the indicated row, to “BARX1/Mem” for the first row (“cat01 fibrob.”). Rows as follows: “cat01 fibrobl.”, fibroblasts from skin sample from cat01; “cat04 FISS”, cells derived from FISS tumor sample from cat04; “cat05 FISS”, cells derived from FISS tumor sample from cat05. (DOCX 44 kb)

Additional file 7: **Figure S2.** Full membrane images for BARX1 and FN1 immunoblots. (A) Anti-BARX1 stained Western with ladder. (B) Same membrane stained for total protein with Pierce Removable Total Protein. (C) Anti-FN1 stained Western with ladder. (B) Same membrane stained for total protein with Pierce Removable Total Protein. (PDF 132 kb)

Additional file 8: **Table S5.** Quantification of protein expression of FN1 in fibroblasts and FISS cells. Columns as follows: “220 kDA FN1”, integrated intensity of the FN1 band at 220 kDa; “40 kDa Memcode”, integrated intensity of the normalization control chain at 40 kDa; “FN1/Mem”, ratio of intensity of the band at 220 kDa to the intensity of the band at 40 kDa, for the indicated row; “FISS/fibrobl”, ratio of “FN1/Mem” for the indicated row, to “FN1/Mem” for the first row (“cat01 fibrob.”). Rows as follows: “cat01 fibrobl.”, fibroblasts from skin sample from cat01; “cat04 FISS”, cells derived from FISS tumor sample from cat04; “cat05 FISS”, cells derived from FISS tumor sample from cat05. (DOCX 38 kb)

## Abbreviations

FISS: feline injection site sarcoma
GSEA: gene set enrichment analysis
PCA: principal component analysis
SCNA: somatic copy number alteration

## Footnotes

1 This included cat genes without human orthologs.
