# Supplementary material for: Elucidating the transcriptional program of feline injection-site sarcoma using a cross-species mRNA-sequencing approach
Source: BMC Cancer. 2019 Apr 4;19:311. doi: 10.1186/s12885-019-5501-z (PMC6449919; doi:10.1186/s12885-019-5501-z)
Supplement: Supplementary file 2 — Supplementary Note 1: This file contains the cDNA sequence used for the qPCR assay design for the gene LATS1. (PDF 42 kb) [file 12885_2019_5501_MOESM2_ESM.pdf]

cDNA sequence used for LATS1 qPCR assay design:

For *LATS1*, a custom TaqMan assay was used based on the submitted sequence:

GAGTACTTCAGAAGTTAATCCACAAATGCTTCAAGATTTGCAAGCTGCTGGA
TTTGATGAGGATATGGTTATACAAGCTCTTCAGAAAACTAACAACAGAAGTA
TAGAGGCAGCAATTGAATTCATTAGTAAAATGAGTTACCAAGATCCTCGCCG
GGAACAGAT.
