# Supplementary figures and images for: Elucidating the transcriptional program of feline injection-site sarcoma using a cross-species mRNA-sequencing approach
Source: BMC Cancer. 2019 Apr 4;19:311. doi: 10.1186/s12885-019-5501-z (PMC6449919; doi:10.1186/s12885-019-5501-z)

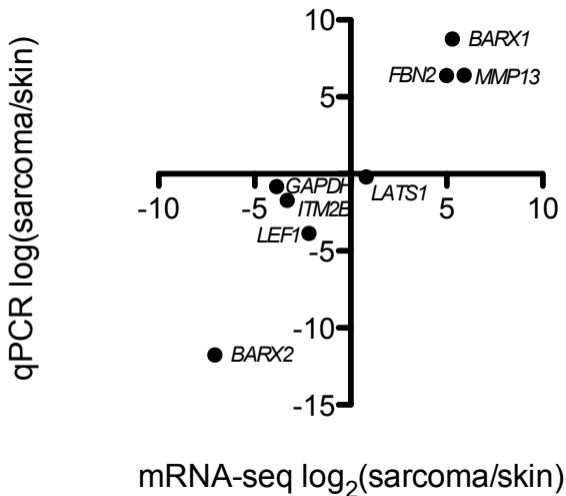

Supplement: Supplementary file 4 — Figure S1. Concordance of qPCR measurements with mRNA-seq measurements of relative gene expression in FISS vs. skin samples. Each mark represents a gene (genes are as described in Table 1) and marks are labeled by official HGNC gene symbol. (PDF 26 kb) [file 12885_2019_5501_MOESM4_ESM.pdf]

A

anti-BARX1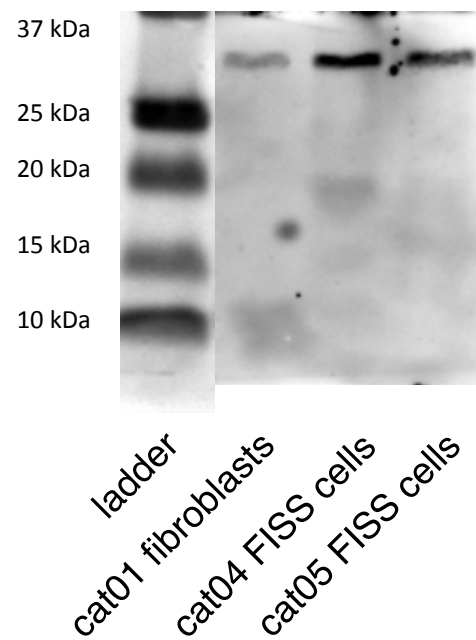

B

Total protein stain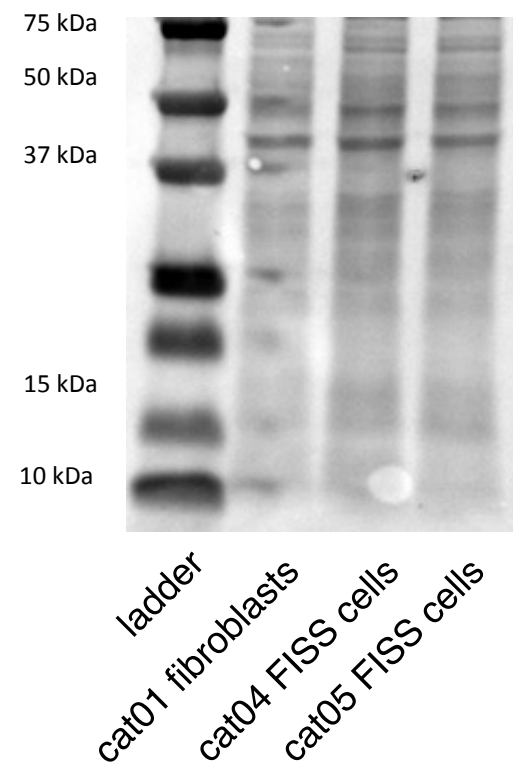

C

anti-FN1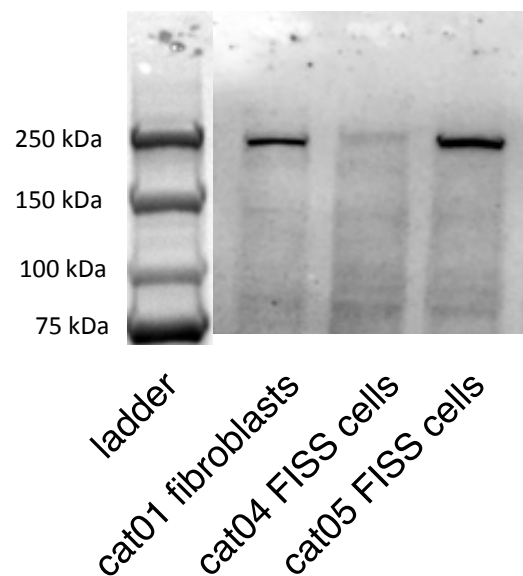

D

Total protein stain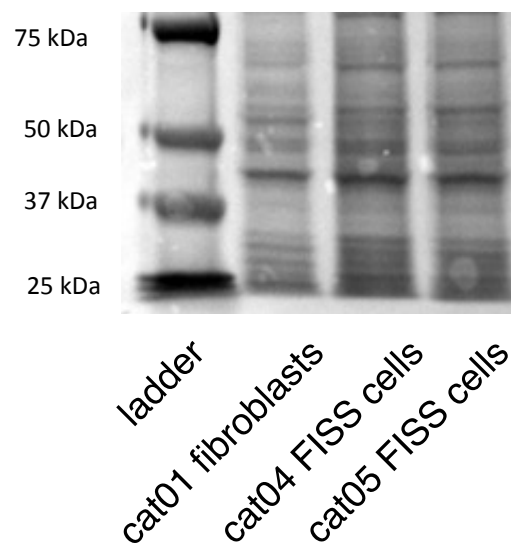

Supplement: Supplementary file 7 — Figure S2. Full membrane images for BARX1 and FN1 immunoblots. (A) Anti-BARX1 stained Western with ladder. (B) Same membrane stained for total protein with Pierce Removable Total Protein. (C) Anti-FN1 stained Western with ladder. (B) Same membrane stained for total protein with Pierce Removable Total Protein. (PDF 132 kb) [file 12885_2019_5501_MOESM7_ESM.pdf]
