# Supplementary material for: Elucidating the transcriptional program of feline injection-site sarcoma using a cross-species mRNA-sequencing approach
Source: BMC Cancer. 2019 Apr 4;19:311. doi: 10.1186/s12885-019-5501-z (PMC6449919; doi:10.1186/s12885-019-5501-z)
Supplement: Supplementary file 5 — Table S3. Regions of coherent up- or down-regulation in sarcoma vs. normal tissue. Each row corresponds to a 10 Mbp region of the cat genome for which the expression levels of the genes within the region are (together) consistently up- or down-regulated in sarcoma tumor tissue vs. normal skin. Columns as follows: Fc chr., cat chromosome; Fc pos. (Mbp), the chromosomal coordinate of the start of the region, in Mbp; Genes within region, the Ensembl gene identifiers of all genes that are annotated within the region; log2(sarcoma/normal), the average of the log2(sarcoma/normal) expression values for all genes in the region; Hs chr., the chromosome of the human genome region that is syntenic to the indicated cat genome region; Hs pos, the coordinates (in the GRCh38 genome assembly) of the human genome region that is syntenic to the indicated cat genome region. (DOCX 128 kb) [file 12885_2019_5501_MOESM5_ESM.docx]

| *Fc*. chr. | *Fc*. pos. (Mbp) | Genes within region | log_2_  (sarcoma/  normal) | *Hs*. chr. | *Hs*. pos.  (Mbp, GRCh38) |
| --- | --- | --- | --- | --- | --- |
| D3 | 50 | ENSFCAG00000011715, ENSFCAG00000025165, ENSFCAG00000011308, ENSFCAG00000027450, ENSFCAG00000030737, ENSFCAG00000028996, ENSFCAG00000031128, ENSFCAG00000011314, ENSFCAG00000023041, ENSFCAG00000014180, ENSFCAG00000014182, ENSFCAG00000026319, ENSFCAG00000024545, ENSFCAG00000011492, ENSFCAG00000024870, ENSFCAG00000023533, ENSFCAG00000031299, ENSFCAG00000029008, ENSFCAG00000027087, ENSFCAG00000022059, ENSFCAG00000000626, ENSFCAG00000025491, ENSFCAG00000008801, ENSFCAG00000008802, ENSFCAG00000008804 | -2.38 | 18 | 20.9–80.3  Recurrently deleted in human sarcoma (*Hs*-18q23) |
| A1 | 60 | ENSFCAG00000027269, ENSFCAG00000016606, ENSFCAG00000016469, ENSFCAG00000016537, ENSFCAG00000016427, ENSFCAG00000016573, ENSFCAG00000016478, ENSFCAG00000022529, ENSFCAG00000025920, ENSFCAG00000026347, ENSFCAG00000029638, ENSFCAG00000028274, ENSFCAG00000031198, ENSFCAG00000027133, ENSFCAG00000028336, ENSFCAG00000015330, ENSFCAG00000022641, ENSFCAG00000025657, ENSFCAG00000014842, ENSFCAG00000010224, ENSFCAG00000026940 | -1.96 | 13 | 52.6–114.3 |
| D3 | 80 | ENSFCAG00000003171, ENSFCAG00000019009, ENSFCAG00000013841, ENSFCAG00000023181, ENSFCAG00000024132, ENSFCAG00000030054, ENSFCAG00000018908, ENSFCAG00000008379, ENSFCAG00000005348, ENSFCAG00000018359, ENSFCAG00000024196, ENSFCAG00000022639, ENSFCAG00000031659, ENSFCAG00000008383, ENSFCAG00000030347, ENSFCAG00000029968, ENSFCAG00000019323, ENSFCAG00000022586, ENSFCAG00000024123, ENSFCAG00000025129, ENSFCAG00000022364, ENSFCAG00000027229, ENSFCAG00000015556, ENSFCAG00000026408, ENSFCAG00000004167, ENSFCAG00000009195 | -1.52 | 18 | 20.9–80.3 |
| C1 | 100 | ENSFCAG00000011474, ENSFCAG00000031382, ENSFCAG00000028868, ENSFCAG00000026455, ENSFCAG00000030661, ENSFCAG00000031847, ENSFCAG00000027086, ENSFCAG00000023617, ENSFCAG00000030198, ENSFCAG00000007777, ENSFCAG00000027476, ENSFCAG00000000349, ENSFCAG00000012909, ENSFCAG00000024477, ENSFCAG00000013367, ENSFCAG00000013360, ENSFCAG00000022065, ENSFCAG00000005410, ENSFCAG00000024838, ENSFCAG00000027331, ENSFCAG00000023368, ENSFCAG00000027797, ENSFCAG00000024806, ENSFCAG00000010103, ENSFCAG00000010112, ENSFCAG00000025258, ENSFCAG00000013934, ENSFCAG00000013939, ENSFCAG00000013937, ENSFCAG00000029933, ENSFCAG00000013940, ENSFCAG00000030083, ENSFCAG00000001702, ENSFCAG00000027224, ENSFCAG00000000754, ENSFCAG00000022764, ENSFCAG00000009655, ENSFCAG00000009658, ENSFCAG00000024612, ENSFCAG00000025753, ENSFCAG00000027874, ENSFCAG00000005555, ENSFCAG00000005558, ENSFCAG00000005560, ENSFCAG00000005563, ENSFCAG00000005564, ENSFCAG00000005566, ENSFCAG00000001861, ENSFCAG00000004072, ENSFCAG00000004073, ENSFCAG00000024744, ENSFCAG00000019369, ENSFCAG00000010979, ENSFCAG00000013923, ENSFCAG00000013569, ENSFCAG00000001966, ENSFCAG00000027370, ENSFCAG00000002483, ENSFCAG00000016604, ENSFCAG00000008456, ENSFCAG00000029217, ENSFCAG00000000301, ENSFCAG00000027063, ENSFCAG00000030987, ENSFCAG00000029228 | -0.93 | 1 | 30.6–120.2 |
| E2 | 10 | ENSFCAG00000007337, ENSFCAG00000023587, ENSFCAG00000007335, ENSFCAG00000007334, ENSFCAG00000030970, ENSFCAG00000030325, ENSFCAG00000022530, ENSFCAG00000007330, ENSFCAG00000016426, ENSFCAG00000031197, ENSFCAG00000030677, ENSFCAG00000007430, ENSFCAG00000007439, ENSFCAG00000023961, ENSFCAG00000002679, ENSFCAG00000002672, ENSFCAG00000004804, ENSFCAG00000027259, ENSFCAG00000023447, ENSFCAG00000026142, ENSFCAG00000010611, ENSFCAG00000031026, ENSFCAG00000029102, ENSFCAG00000000964, ENSFCAG00000000967, ENSFCAG00000000969, ENSFCAG00000018228, ENSFCAG00000002112, ENSFCAG00000030201, ENSFCAG00000002117, ENSFCAG00000009561, ENSFCAG00000028840, ENSFCAG00000009559, ENSFCAG00000009558, ENSFCAG00000031825, ENSFCAG00000030946, ENSFCAG00000030957, ENSFCAG00000031691, ENSFCAG00000026255, ENSFCAG00000001105, ENSFCAG00000001104, ENSFCAG00000005230, ENSFCAG00000005229, ENSFCAG00000021940, ENSFCAG00000005222, ENSFCAG00000027744, ENSFCAG00000025344, ENSFCAG00000025558, ENSFCAG00000025352, ENSFCAG00000029888, ENSFCAG00000008126, ENSFCAG00000008125, ENSFCAG00000008129, ENSFCAG00000008128, ENSFCAG00000022250, ENSFCAG00000022743, ENSFCAG00000012737, ENSFCAG00000012741, ENSFCAG00000012742, ENSFCAG00000012745, ENSFCAG00000002361, ENSFCAG00000005440, ENSFCAG00000027052, ENSFCAG00000005437, ENSFCAG00000005436, ENSFCAG00000019378, ENSFCAG00000005057, ENSFCAG00000027930, ENSFCAG00000027321, ENSFCAG00000018807, ENSFCAG00000018144, ENSFCAG00000004467, ENSFCAG00000001032, ENSFCAG00000001031, ENSFCAG00000007031, ENSFCAG00000030814, ENSFCAG00000001029, ENSFCAG00000001027, ENSFCAG00000012180, ENSFCAG00000012179, ENSFCAG00000012178, ENSFCAG00000030745, ENSFCAG00000018512, ENSFCAG00000003026, ENSFCAG00000003021, ENSFCAG00000024426, ENSFCAG00000010743, ENSFCAG00000010740, ENSFCAG00000010738, ENSFCAG00000030723, ENSFCAG00000031417, ENSFCAG00000005132, ENSFCAG00000024364, ENSFCAG00000012176, ENSFCAG00000030831, ENSFCAG00000027779, ENSFCAG00000003187, ENSFCAG00000003185, ENSFCAG00000003184, ENSFCAG00000003183, ENSFCAG00000003182, ENSFCAG00000003181, ENSFCAG00000009930, ENSFCAG00000000833, ENSFCAG00000000829, ENSFCAG00000000827, ENSFCAG00000000824, ENSFCAG00000014834, ENSFCAG00000026671, ENSFCAG00000014490, ENSFCAG00000027169, ENSFCAG00000026405, ENSFCAG00000031886, ENSFCAG00000028628, ENSFCAG00000029459, ENSFCAG00000025455, ENSFCAG00000023250, ENSFCAG00000029001, ENSFCAG00000024340, ENSFCAG00000023086, ENSFCAG00000026545, ENSFCAG00000025315, ENSFCAG00000031888, ENSFCAG00000014155, ENSFCAG00000026052, ENSFCAG00000014136, ENSFCAG00000014135, ENSFCAG00000022848, ENSFCAG00000006619, ENSFCAG00000006617, ENSFCAG00000029538, ENSFCAG00000027180, ENSFCAG00000024555, ENSFCAG00000018381, ENSFCAG00000023217, ENSFCAG00000006386, ENSFCAG00000006383, ENSFCAG00000006374, ENSFCAG00000023717, ENSFCAG00000006367, ENSFCAG00000006507, ENSFCAG00000025809, ENSFCAG00000008892, ENSFCAG00000008889, ENSFCAG00000023692, ENSFCAG00000022080, ENSFCAG00000025865, ENSFCAG00000007976, ENSFCAG00000014921, ENSFCAG00000014916, ENSFCAG00000014915, ENSFCAG00000027289, ENSFCAG00000014914, ENSFCAG00000028692, ENSFCAG00000002328, ENSFCAG00000017581 | -0.75 | 19 | 28.1–58.6 |
| D1 | 60 | ENSFCAG00000007938, ENSFCAG00000028884, ENSFCAG00000005033, ENSFCAG00000005031, ENSFCAG00000028250, ENSFCAG00000023834, ENSFCAG00000002870, ENSFCAG00000031954, ENSFCAG00000026356, ENSFCAG00000013192, ENSFCAG00000013190, ENSFCAG00000016681, ENSFCAG00000005325, ENSFCAG00000005324, ENSFCAG00000005321, ENSFCAG00000005320, ENSFCAG00000005317, ENSFCAG00000026180, ENSFCAG00000031838, ENSFCAG00000003743, ENSFCAG00000007813, ENSFCAG00000024506, ENSFCAG00000007815, ENSFCAG00000018739, ENSFCAG00000026680, ENSFCAG00000027390, ENSFCAG00000021997, ENSFCAG00000026827, ENSFCAG00000027415, ENSFCAG00000005372, ENSFCAG00000015315, ENSFCAG00000015318, ENSFCAG00000015319, ENSFCAG00000010240, ENSFCAG00000015320, ENSFCAG00000025524, ENSFCAG00000028144, ENSFCAG00000010990, ENSFCAG00000028022, ENSFCAG00000010992, ENSFCAG00000025375, ENSFCAG00000025216, ENSFCAG00000001336, ENSFCAG00000002355, ENSFCAG00000027015, ENSFCAG00000026403, ENSFCAG00000030797, ENSFCAG00000031138, ENSFCAG00000027921, ENSFCAG00000027443, ENSFCAG00000031616, ENSFCAG00000029784, ENSFCAG00000025293, ENSFCAG00000022132, ENSFCAG00000011568, ENSFCAG00000023121, ENSFCAG00000025742, ENSFCAG00000027369, ENSFCAG00000011572, ENSFCAG00000025819, ENSFCAG00000031162, ENSFCAG00000010733, ENSFCAG00000028634, ENSFCAG00000030103, ENSFCAG00000022225, ENSFCAG00000010735, ENSFCAG00000029152, ENSFCAG00000023095, ENSFCAG00000028297, ENSFCAG00000024740, ENSFCAG00000029477, ENSFCAG00000024745, ENSFCAG00000030801, ENSFCAG00000023295, ENSFCAG00000024274, ENSFCAG00000030289, ENSFCAG00000031334, ENSFCAG00000026886, ENSFCAG00000023177, ENSFCAG00000031645, ENSFCAG00000026984, ENSFCAG00000000718, ENSFCAG00000026954, ENSFCAG00000025048, ENSFCAG00000028751, ENSFCAG00000023093, ENSFCAG00000025719, ENSFCAG00000026738, ENSFCAG00000022347, ENSFCAG00000031727, ENSFCAG00000007878, ENSFCAG00000027630, ENSFCAG00000004625, ENSFCAG00000023147, ENSFCAG00000023578, ENSFCAG00000011695, ENSFCAG00000026173, ENSFCAG00000030000, ENSFCAG00000027196, ENSFCAG00000004633, ENSFCAG00000024710, ENSFCAG00000015388, ENSFCAG00000022056, ENSFCAG00000031950, ENSFCAG00000014891, ENSFCAG00000006190, ENSFCAG00000012347, ENSFCAG00000028512, ENSFCAG00000011486, ENSFCAG00000005217, ENSFCAG00000028968, ENSFCAG00000019083, ENSFCAG00000029053, ENSFCAG00000023263, ENSFCAG00000028041, ENSFCAG00000026973, ENSFCAG00000000730, ENSFCAG00000008411, ENSFCAG00000006543, ENSFCAG00000030327, ENSFCAG00000002342, ENSFCAG00000023566, ENSFCAG00000003774, ENSFCAG00000029140, ENSFCAG00000002289, ENSFCAG00000000400, ENSFCAG00000000407 | 0.50 | 11 | 71.9–101.4 |
| D4 | 0 | ENSFCAG00000029042, ENSFCAG00000010215, ENSFCAG00000000820, ENSFCAG00000027526, ENSFCAG00000015134, ENSFCAG00000021928, ENSFCAG00000015141, ENSFCAG00000015143, ENSFCAG00000009016, ENSFCAG00000009018, ENSFCAG00000009022, ENSFCAG00000016624, ENSFCAG00000016529, ENSFCAG00000016498, ENSFCAG00000030763, ENSFCAG00000007197, ENSFCAG00000029322, ENSFCAG00000004210, ENSFCAG00000022286, ENSFCAG00000004214, ENSFCAG00000022749, ENSFCAG00000014214, ENSFCAG00000021943, ENSFCAG00000021894, ENSFCAG00000024643, ENSFCAG00000014912, ENSFCAG00000023196, ENSFCAG00000011471, ENSFCAG00000022543, ENSFCAG00000028063, ENSFCAG00000002369, ENSFCAG00000026636, ENSFCAG00000002365, ENSFCAG00000012950, ENSFCAG00000031060, ENSFCAG00000022615, ENSFCAG00000013260, ENSFCAG00000021117 | 0.64 | 9 | 92.2–94.5 |
| B3 | 140 | ENSFCAG00000006777, ENSFCAG00000006779, ENSFCAG00000027708, ENSFCAG00000006783, ENSFCAG00000006784, ENSFCAG00000008254, ENSFCAG00000029179, ENSFCAG00000021945, ENSFCAG00000001352, ENSFCAG00000004659, ENSFCAG00000025054, ENSFCAG00000016587, ENSFCAG00000024327, ENSFCAG00000024973, ENSFCAG00000010000, ENSFCAG00000031226, ENSFCAG00000000458, ENSFCAG00000016668, ENSFCAG00000016603, ENSFCAG00000016653, ENSFCAG00000016500, ENSFCAG00000016517, ENSFCAG00000016649, ENSFCAG00000018318, ENSFCAG00000016491, ENSFCAG00000016433, ENSFCAG00000016613, ENSFCAG00000016667, ENSFCAG00000016633, ENSFCAG00000018219, ENSFCAG00000027739, ENSFCAG00000016494, ENSFCAG00000016448, ENSFCAG00000016424, ENSFCAG00000021358, ENSFCAG00000023710, ENSFCAG00000016622, ENSFCAG00000025326, ENSFCAG00000018345, ENSFCAG00000025405, ENSFCAG00000028977, ENSFCAG00000016520, ENSFCAG00000019388, ENSFCAG00000022279, ENSFCAG00000016551, ENSFCAG00000019361, ENSFCAG00000016549, ENSFCAG00000016572, ENSFCAG00000016492, ENSFCAG00000018014, ENSFCAG00000016605, ENSFCAG00000016497, ENSFCAG00000030135, ENSFCAG00000016590, ENSFCAG00000016470, ENSFCAG00000016687, ENSFCAG00000016531, ENSFCAG00000016566, ENSFCAG00000027592, ENSFCAG00000022410, ENSFCAG00000029926, ENSFCAG00000016464, ENSFCAG00000030240, ENSFCAG00000016435, ENSFCAG00000031258, ENSFCAG00000015428, ENSFCAG00000023830, ENSFCAG00000008938, ENSFCAG00000000815, ENSFCAG00000022378, ENSFCAG00000023382, ENSFCAG00000004651, ENSFCAG00000010430, ENSFCAG00000010003, ENSFCAG00000025432, ENSFCAG00000010009, ENSFCAG00000026698, ENSFCAG00000002239, ENSFCAG00000026424, ENSFCAG00000022667, ENSFCAG00000007949, ENSFCAG00000030816, ENSFCAG00000027939, ENSFCAG00000014599, ENSFCAG00000021907, ENSFCAG00000013807, ENSFCAG00000024828, ENSFCAG00000028169, ENSFCAG00000023037, ENSFCAG00000028425, ENSFCAG00000004939, ENSFCAG00000004946, ENSFCAG00000004953, ENSFCAG00000000681, ENSFCAG00000011100, ENSFCAG00000000683, ENSFCAG00000029656, ENSFCAG00000030580, ENSFCAG00000000688, ENSFCAG00000000689, ENSFCAG00000016258, ENSFCAG00000028779 | 0.75 | 14 | 40.3–106 |
| C1 | 70 | ENSFCAG00000008789, ENSFCAG00000025546, ENSFCAG00000022598, ENSFCAG00000011197, ENSFCAG00000031965, ENSFCAG00000008210, ENSFCAG00000031867, ENSFCAG00000018343, ENSFCAG00000030426, ENSFCAG00000028907, ENSFCAG00000028517, ENSFCAG00000008006, ENSFCAG00000011948, ENSFCAG00000024813, ENSFCAG00000004292, ENSFCAG00000030043, ENSFCAG00000012994, ENSFCAG00000031834, ENSFCAG00000030586, ENSFCAG00000026166, ENSFCAG00000029587, ENSFCAG00000024034, ENSFCAG00000001592, ENSFCAG00000007985, ENSFCAG00000027961, ENSFCAG00000000151, ENSFCAG00000027703, ENSFCAG00000026327, ENSFCAG00000024680, ENSFCAG00000015025, ENSFCAG00000015028, ENSFCAG00000012458, ENSFCAG00000031485, ENSFCAG00000012766, ENSFCAG00000018347, ENSFCAG00000015094, ENSFCAG00000022261, ENSFCAG00000028155 | 0.81 | 1 | 30.6–120.2 |
