# Supplementary material for: Elucidating the transcriptional program of feline injection-site sarcoma using a cross-species mRNA-sequencing approach
Source: BMC Cancer. 2019 Apr 4;19:311. doi: 10.1186/s12885-019-5501-z (PMC6449919; doi:10.1186/s12885-019-5501-z)
Supplement: Supplementary file 6 — Table S4. Quantification of protein expression of BARX1 in fibroblasts and FISS cells. Columns as follows: “27 kDA BARX1”, integrated intensity of the BARX1 band at 27 kDa; “40 kDa Memcode”, integrated intensity of the normalization control chain at 40 kDa; “BARX1/Mem”, ratio of intensity of the band at 27 kDa to the intensity of the band at 40 kDa, for the indicated row; “FISS/fibrobl”, ratio of “BARX1/Mem” for the indicated row, to “BARX1/Mem” for the first row (“cat01 fibrob.”). Rows as follows: “cat01 fibrobl.”, fibroblasts from skin sample from cat01; “cat04 FISS”, cells derived from FISS tumor sample from cat04; “cat05 FISS”, cells derived from FISS tumor sample from cat05. (DOCX 44 kb) [file 12885_2019_5501_MOESM6_ESM.docx]

|  | 27 kDa | 40 kDa |  |  |
| --- | --- | --- | --- | --- |
|  | BARX1 | Memcode | BARX1/Mem | FISS/fibrobl. |
| cat01 fibrobl. | 3086955 | 3278875 | 0.9415 |  |
| cat04 FISS | 7261404 | 2560906 | 2.8355 | 3.01 |
| cat05 FISS | 7243003 | 2512803 | 2.8824 | 3.06 |
