# Supplementary material for: Elucidating the transcriptional program of feline injection-site sarcoma using a cross-species mRNA-sequencing approach
Source: BMC Cancer. 2019 Apr 4;19:311. doi: 10.1186/s12885-019-5501-z (PMC6449919; doi:10.1186/s12885-019-5501-z)
Supplement: Supplementary file 8 — Table S5. Quantification of protein expression of FN1 in fibroblasts and FISS cells. Columns as follows: “220 kDA FN1”, integrated intensity of the FN1 band at 220 kDa; “40 kDa Memcode”, integrated intensity of the normalization control chain at 40 kDa; “FN1/Mem”, ratio of intensity of the band at 220 kDa to the intensity of the band at 40 kDa, for the indicated row; “FISS/fibrobl”, ratio of “FN1/Mem” for the indicated row, to “FN1/Mem” for the first row (“cat01 fibrob.”). Rows as follows: “cat01 fibrobl.”, fibroblasts from skin sample from cat01; “cat04 FISS”, cells derived from FISS tumor sample from cat04; “cat05 FISS”, cells derived from FISS tumor sample from cat05. (DOCX 38 kb) [file 12885_2019_5501_MOESM8_ESM.docx]

|  | 220 kDa | 40 kDa |  |  |
| --- | --- | --- | --- | --- |
|  | FN1 | Memcode | FN1/Mem | FISS/fibrobl. |
| cat01 fibrobl. | 19468061 | 4164745 | 4.6745 |  |
| cat05 FISS | 33377082 | 3358215 | 9.9389 | 2.13 |
